# Prediction of radiosensitivity and radiocurability using a novel supervised artificial neural network

**DOI:** 10.1186/s12885-022-10339-3

**Published:** 2022-12-01

**Authors:** Zihang Zeng, Maoling Luo, Yangyi Li, Jiali Li, Zhengrong Huang, Yuxin Zeng, Yu Yuan, Mengqin Wang, Yuying Liu, Yan Gong, Conghua Xie

**Affiliations:** 1 Department of Radiation and Medical Oncology, Zhongnan Hospital of Wuhan University, 169 Donghu Road, Wuhan, 430071 Hubei China; 2 Department of Biological Repositories, Zhongnan Hospital of Wuhan University, 169 Donghu Road, Wuhan, 430071 Hubei China; 3 Tumor Precision Diagnosis and Treatment Technology and Translational Medicine, Hubei Engineering Research Center, Zhongnan Hospital of Wuhan University, Wuhan, 430071 Hubei China; 4 Hubei Key Laboratory of Tumor Biological Behaviors, Zhongnan Hospital of Wuhan University, Wuhan, 430071 Hubei China; 5 Hubei Cancer Clinical Study Center, Zhongnan Hospital of Wuhan University, Wuhan, 430071 Hubei China

**Keywords:** Computational Biology, High-Throughput Sequencing, Multi-Omics, Neural Network Models, Radiosensitivity

## Abstract

**Background:**

Radiotherapy has been widely used to treat various cancers, but its efficacy depends on the individual involved. Traditional gene-based machine-learning models have been widely used to predict radiosensitivity. However, there is still a lack of emerging powerful models, artificial neural networks (ANN), in the practice of gene-based radiosensitivity prediction. In addition, ANN may overfit and learn biologically irrelevant features.

**Methods:**

We developed a novel ANN with Selective Connection based on Gene Patterns (namely ANN-SCGP) to predict radiosensitivity and radiocurability. We creatively used gene patterns (gene similarity or gene interaction information) to control the "on–off" of the first layer of weights, enabling the low-dimensional features to learn the gene pattern information. ANN-SCGP was trained and tested in 82 cell lines and 1,101 patients from the 11 pan-cancer cohorts.

**Results:**

For survival fraction at 2 Gy, the root mean squared errors (RMSE) of prediction in ANN-SCGP was the smallest among all algorithms (mean RMSE: 0.1587–0.1654). For radiocurability, ANN-SCGP achieved the first and second largest C-index in the 12/20 and 4/20 tests, respectively. The low dimensional output of ANN-SCGP reproduced the patterns of gene similarity. Moreover, the pan-cancer analysis indicated that immune signals and DNA damage responses were associated with radiocurability.

**Conclusions:**

As a model including gene pattern information, ANN-SCGP had superior prediction abilities than traditional models. Our work provided novel insights into radiosensitivity and radiocurability.

**Supplementary Information:**

The online version contains supplementary material available at 10.1186/s12885-022-10339-3.

## Introduction

Radiotherapy (RT) has been widely used in various tumors, but radioresistance is the leading cause of recurrent local lesions and poor prognosis after RT [1]. Radioresistance involves multiple molecules and pathways in tumor cells and the tumor microenvironment [2]. The estimation of radiosensitivity will benefit the prediction of clinical response, which is challenging due to the biological heterogeneity of tumors.

With the coming of the post-genome era, large-scale omics data provide opportunities to characterize the intrinsic tumor profiles of individual patients [3]. Recently, multiple studies demonstrated the effectiveness of gene-based models in predicting radiosensitivity and prognosis for RT patients (i.e., radiocurability). Commonly, the linear model is performed on selected gene signatures to obtain a linear weighted model of genes to predict radiosensitivity. Scott et al. used the 10-gene rank-based linear regression (radiation-sensitivity index, RSI) to derive the genomic-adjusted radiation dose [4], which was widely used in radiation-related study [5]. Liu et al. [6] and Xu et al. [7] also verified the performance of the gene linear weighted model in predicting radiosensitivity. However, in a comparative study, Manem et al. found that regressions with regularization terms were better predictors of survival fraction at 2 Gy (SF2) than simple linear regressions [8]. A large number of regularization regression models (e.g. LASSO regression) have been demonstrated to be effective [9–14]. Other gene-based machine-learning algorithms such as random forest, partial least squares, and SVM could also successfully predict radiosensitivity and radiocurability [15, 16]. Moreover, the k-top scoring pairs algorithms [17] and ensemble-based classifiers [18] are developed to predict radioresistance, however, these methods are focused on the classification task. A summary table of previous gene model studies on radiosensitivity is shown in Supplementary Table 1. One main gap in the field of gene-based radiosensitivity models is the lack of application of emerging algorithms (i.e., artificial neural networks, ANN), and the lack of a pan-cancer comparison of various traditional machine learning algorithms and ANN.

Traditional linear models could not characterize nonlinear patterns between genes and radiosensitivity. As an algorithm with strong nonlinear fitting ability, ANN has been widely used in biological discovery. Le et al. developed a novel convolutional neural network-based method to identify N6-methyladenine [19]. Tng et al. recognized the histone lysine crotonylation using a novel recurrent neural network [20]. In prognosis prediction, Luo et al. trained a gene expression-based fully connected ANN [21]. These works demonstrated the exciting prospects of ANN in bioinformatics. However, there is still a gap between ANN and biological significance. Specifically, ANN has the potential to be overfitted and learn biologically irrelevant deep features. Furthermore, ANN's biological interpretability is also unsatisfactory.

The above 2 gaps motivated us to design ANN models with more biological interpretability, and benchmark traditional machine learning algorithms and ANN to predict radiosensitivity. It is generally accepted that genes perform biological functions through regulatory networks, and similar genes are more likely to be linked to similar functions, which inspires us to develop a novel supervised ANN with Selective Connection Based on Gene Patterns (namely ANN-SCGP) to predict radiosensitivity and radiocurability. The main idea of ANN-SCGP is to map gene similarity or gene connection information to the weight of the first layer to control the "on–off" of the weight, the deep node of the first hidden layer selectively receives signals from similar genes, thus artificially giving biological interpretability to the deep node of the first hidden layer. In Sect. 3.3.4, we confirmed that the output of the first hidden layer can reproduce gene pattern information, which can promote the ANN learning of biologically relevant low-dimensional features. In this study, we first proposed a novel workflow to identify radiation-related signatures (RRS). Next, we compared the linear regression model (LM), LASSO regression, Elastic net regression, support vector machine (SVM), Cox proportional hazard (CPH) model, random forest (RF), random survival forest (RSF), partial least squares (PLS), RSI, and fully-connected ANN in pan-cancer datasets. ANN-SCGP had superior predictive power on radiosensitivity and radiocurability.

## Materials and methods

### Data collection and standardization

The pan-cancer omics-data of the 82 cell lines were collected from the Cancer Cell Line Encyclopedia (CCLE) and Genomics of Drug Sensitivity in Cancer (GDSC) datasets for SF2 prediction. The omics data and clinical annotations of the 1,101 patients were collected from the Cancer Genome Atlas (TCGA) and GSE68465 datasets for survival prediction. In the SF2 prediction task, the ground truth of SF2 values in cell lines of CCLE and GDSC was accessed from previous laboratory experiments. In the overall survival (OS) prediction task, the ground truth of survival times was obtained from follow-up information in the TCGA database. OS of patients with RT was defined as radiocurability. The detail of data collection and standardization were shown in.

### ANN-SCGP: Artificial neural network with selective connection based on gene patterns

ANN-SCGP was a feedforward ANN model having a selective connection based on gene patterns from the input layer to layer 1 (L1) optimized by the gradient descent algorithm. The other layers of the model consisted of full connections. We used R languages (version 3.6.1) to build this approach which also depended on R NMF package (version 0.23.0), R survival package (version 3.2.11), R philentropy package (version 0.5.0), and R reshape2 package (version 1.4.4). Our ANN-SCGP R package is available in the GitHub repository: https://github.com/ZengZihang/ANNSCGP.

#### Normalization of the input matrix

The gene-patient omics matrix was used as input to ANN-SCGP. The input matrix (n features * m samples) should be normalized to [0, 1] by sample. For Expression and Methylation, the omics data of single sample were normalized by rank-based method to correct batch effect:

1 $$\frac{\mathrm{rank}\left(\mathrm{x}\right)}{\mathrm{Nx}}$$

where the rank(x) was ascending sequencing value of gene x, and Nx is the total number of genes. For copy number alteration (CNA), we linearly transformed discrete values (-2, -1, 0, 1, 2) to range from 0 to 1 (0, 0.25, 0.5, 0.75, 1). Mutation data were binary values from 0 to 1.

#### Initialization of weight and bias

We used Xavier initialization [22] in this study:

2 $${\mathrm{W}}_{\mathrm{l}}\sim \mathrm{N}\left(\upmu =0,{\upsigma }^{2}=\frac{2}{{\mathrm{n}}_{\mathrm{l}}+{\mathrm{n}}_{\mathrm{l}+1}}\right)$$

3 $${\mathrm{B}}_{\mathrm{l}}\sim 0$$

where the ${\mathrm{W}}_{\mathrm{l}}$ is the weight from layer l to l + 1, and ${\mathrm{n}}_{\mathrm{l}}$ represents the number of nodes in layer l. The ${\mathrm{B}}_{\mathrm{l}}$ is the bias term from layer l to l + 1.

#### Selectively connected matrix (SCM) of ANN-SCGP

We constructed a binary SCM (0 is connected; 1 is non-connected) that controlled the on–off state of the L1 weights. To enable ANN to learn gene pattern features, we mapped gene similarity or gene interaction information to SCM. There were 2 strategies to build SCM: ANN-SCGP based on non-negative matrix factorization (NMF) and autoencoder.

When there was no prior gene interaction matrix, we used the NMF algorithm [23] for the input matrix to get a low dimensional matrix whose dimension was equal to the L1 weights. This low-dimensional matrix was transformed into the binary matrix as SCM based on cut-off values. The number of zeros in SCM was defined as sparsity. The sparsity was a hyperparameter that can be chosen according to Pearson's correlation of Euclidean distance of all gene paired between the input and the L1 weight matrix. In this study, we chose the largest possible sparsity that did not cause the above correlation coefficients to decrease rapidly. The purpose of this step was to make the gene pattern of SCM similar to that in the input matrix. Therefore, similar genes were linked to the similar deep nodes in the first hidden layer.

When there was a prior gene interaction matrix, we trained an autoencoder model with Relu activation fitted input matrix. The lowest dimensional hidden layer of autoencoder was transformed to SCM by a set sparsity threshold. The autoencoder was trained by our R ANN-SCGP package.

#### Activation functions

To avoid gradient explosion and vanishing traps, we used SELU activation in all hidden: layers:

4 $$\mathrm{SELU}\left(\mathrm{x}\right)=\uplambda \left\{\begin{array}{c}x \ \ if\ \ x>0\\ \alpha {\mathrm{e}}^{\mathrm{x}}-\alpha \ \ if\ \ x\le 0\end{array}\right.$$

where the $\uplambda$ is 1.05070098736, and $\mathrm{\alpha }$ is 1.67326324235 according to Klambauer's study [24]. For the output layer, we used Sigmoid activation for SF2 fitting and exp activation ${\mathrm{e}}^{\mathrm{x}}$ for survival fitting.

#### Cost functions

Since SF2 was a continuous variable, we used a quadratic cost function to fit SF2:

5 $${\mathrm{J}}_{\uptheta }=\frac{1}{2\mathrm{m}}\sum_{\mathrm{i}=1}^{\mathrm{m}}{({\mathrm{h}}_{\uptheta }\left({\mathrm{x}}_{\mathrm{i}}\right)-{\mathrm{y}}_{\mathrm{i}})}^{2}$$

where m is the number of samples, ${\mathrm{h}}_{\uptheta }\left({\mathrm{x}}_{\mathrm{i}}\right)$ is the output of sample i, and ${\mathrm{y}}_{\mathrm{i}}$ represents SF2 of sample i. Due to the comparison with other non-regularization algorithms, the cost function of this study did not add regularization term. The OS was the time-event variable. Classic cost function of survival data was negative log partial likelihood cost (NLPL):

6 $${\mathrm{J}}_{\mathrm{\theta i}}=-\sum_{\mathrm{i};{\mathrm{E}}_{\mathrm{i}}=1}^{ }\left({\mathrm{h}}_{\uptheta }\left({\mathrm{x}}_{\mathrm{i}}\right)-\mathrm{log}\sum_{\mathrm{j}\in \mathrm{R}\left({\mathrm{T}}_{\mathrm{i}}\right)}{\mathrm{e}}^{{\mathrm{h}}_{\uptheta }\left({\mathrm{x}}_{\mathrm{i}}\right)}\right)$$

where ${\mathrm{T}}_{\mathrm{i}}$ is survival time or the last follow-up time, ${\mathrm{E}}_{\mathrm{i}}$ represents whether patient i observed the event (event = 1; censored = 0), $\mathrm{R}({\mathrm{T}}_{\mathrm{i}})$ is the set of patients at risk of failure at ${\mathrm{T}}_{\mathrm{i}}$. To reduce the computational consumption, we proposed partial quadratic cost (PQC) for survival fitting:

7 $${\mathrm{J}}_{\mathrm{\theta i}}=\frac{1}{2\mathrm{m}}\sum_{\mathrm{i};{\mathrm{E}}_{\mathrm{i}}=1|{\mathrm{h}}_{\uptheta }\left({\mathrm{x}}_{\mathrm{i}}\right)<{\mathrm{T}}_{\mathrm{i}}}^{\mathrm{m}}{({\mathrm{h}}_{\uptheta }\left({\mathrm{x}}_{\mathrm{i}}\right)-{\mathrm{T}}_{\mathrm{i}})}^{2}$$

Compared with NLPL, PQC was faster without loss of accuracy in both training and testing.

#### Evaluation of node importance

To evaluate the importance of gene nodes in the ANN-SCGP model, we performed occlusion tests which were performed at the single-gene level. For the occlusion test of gene_i_, the weight from gene_i_ to all deep nodes was set as zero in weight_1_. Next, we compared the fitting performance before and after occlusion. The indexes of fitting performance were root mean squared error (RMSE) for SF2 fitting and C-index for OS fitting:

8 $${\mathrm{occlusion}}_{\mathrm{i}}=\mathrm{RMSE}\left({\mathrm{h}}_{\uptheta }\left({\mathrm{x}}_{\mathrm{i}},{\mathrm{W}0}_{\mathrm{i},1}\right),\mathrm{SF}2\right)-\mathrm{RMSE}\left({\mathrm{h}}_{\uptheta }\left({\mathrm{x}}_{\mathrm{i}},{\mathrm{W}}_{\mathrm{i},1}\right),\mathrm{SF}2\right)$$

or

9 $${\mathrm{occlusion}}_{\mathrm{i}}=\mathrm{C}\_\mathrm{index}\left({\mathrm{h}}_{\uptheta }\left({\mathrm{x}}_{\mathrm{i}},{\mathrm{W}}_{\mathrm{i},1}\right),\mathrm{OS}\right)-\mathrm{C}\_\mathrm{index}\left({\mathrm{h}}_{\uptheta }\left({\mathrm{x}}_{\mathrm{i}},{\mathrm{W}0}_{\mathrm{i},1}\right),\mathrm{OS}\right)$$

where ${\mathrm{W}0}_{\mathrm{i},1}$ is the weight_1_ whose value from gene_i_ to all deep nodes was set as zero.

### Other methods

We developed a novel 3-step method to identify radiation-related signatures (RRS). In addition, other machine learning and bioinformatics algorithms were shown in Additional file 1.

### Statistical analysis

All statistical analysis was implemented by R 3.6.1. Survival analysis was performed by R survival and visualized by R survminer packages. Receiver operating curve (ROC) analysis and area under the curve (AUC) calculation were based on pROC packages [25]. C-index was defined as the proportion of consistent sample pairs among all available sample pairs. R stats package was used to perform correlation analysis, chi-square, Fisher's test, T-test, analysis of variance, Kruskal–Wallis, and Wilcoxon tests. Beta regression was performed by R betareg package []. Multiple tests were corrected by Benjamini and Hochberg method [27]. The P-value less than 0.05 was considered statistical significance in hypothesis tests. All the *P* values were two-sided. The significant level of false discovery rate q value was also 0.05.

## Results

### Recognition of radiation-related signatures (RRS)

**Fig. 1:**
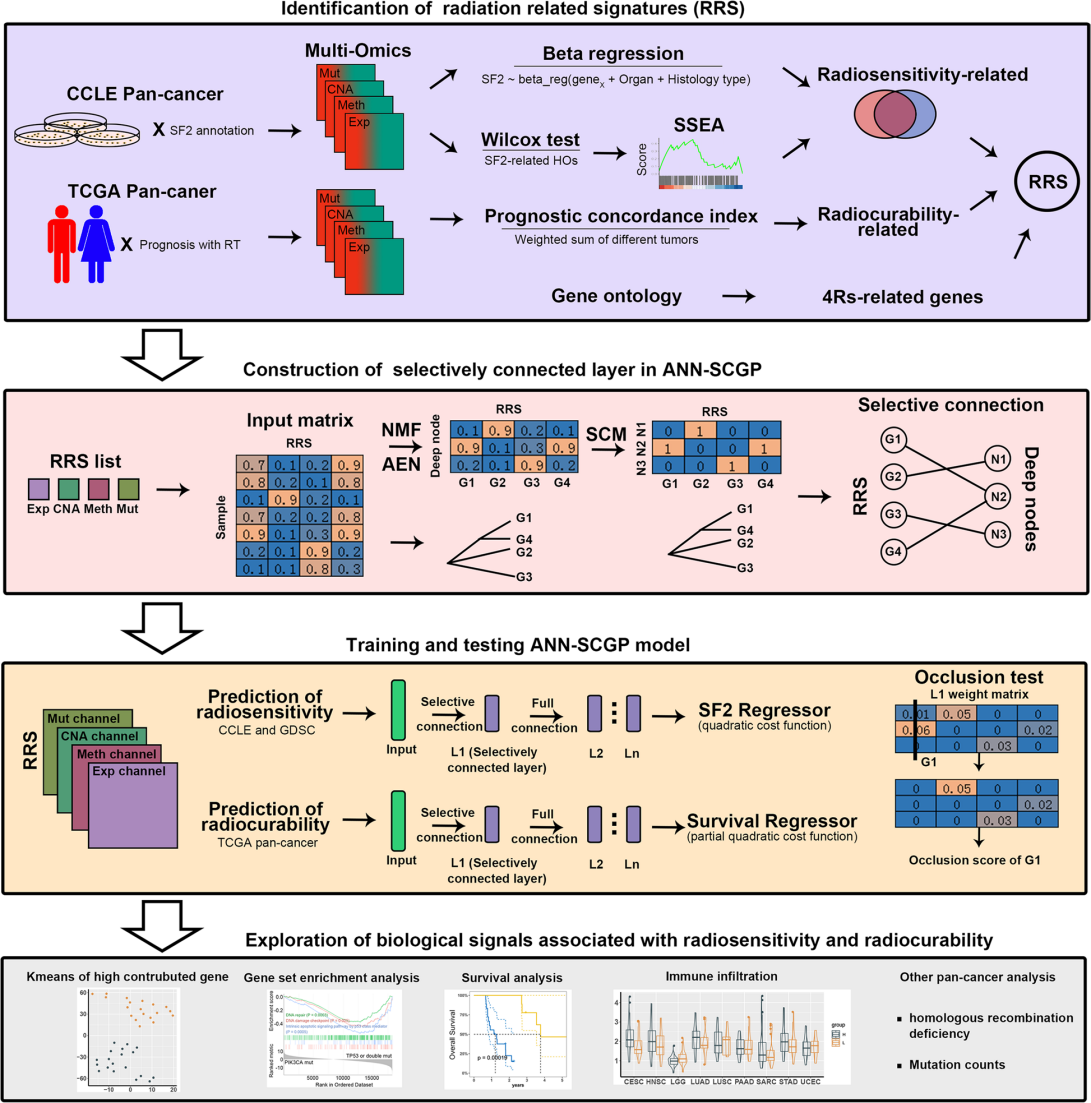
Graphical abstract. NMF, non-negative matrix factorization; AEN, autoencoder; Exp, expression; Meth, methylation; CNA, copy number alteration; Mut, mutation; SF2, survival fraction at 2 Gy; SSEA, Sample set enrichment analysis; L1, the first hidden layer; L2, the second hidden layer

The study design was shown in Fig. 1. We first identified multi-omics RRS as input features of ANN-SCGP through a novel 3-step workflow (Supplementary Fig. 1, see Additional file 1). Finally, the 288 genes were defined as RRS (Supplementary Fig. 2), including 116 expression (Exp) signatures (ANGPT2, CCNA2, CDK6, CHEK1, DNMT3A et al.), 48 methylation (Meth) signatures (CDC20, WNT2B, CDK6, CDK18, DNMT3A et al.), 106 CNA signatures (HDAC9, TP73, TWIST1, CDK11A, CDK11B et al.), and 18 mutation (Mut) signatures (ERBB2, ATRX, NOTCH1, SETX, HSPG2 et al.) (Supplementary Table 2). Our RRS were enriched in a list of genes in the literature-based radiosensitivity database dbCRSR [28] (fisher *p* < 0.00001).

### ANN-SCGP overview

Next, we extracted RRS consisting of Exp, Meth, CNA, and Mut channels linearly normalized to [0, 1] as ANN-SCGP inputs. Unlike the conventional ANN models, ANN-SCGP employed a novel structure that included the SCM as the weight from the input to the first layer based on gene similarity or pre-defined gene interaction networks (see Sect. 2.2.3).

**Fig. 2:**
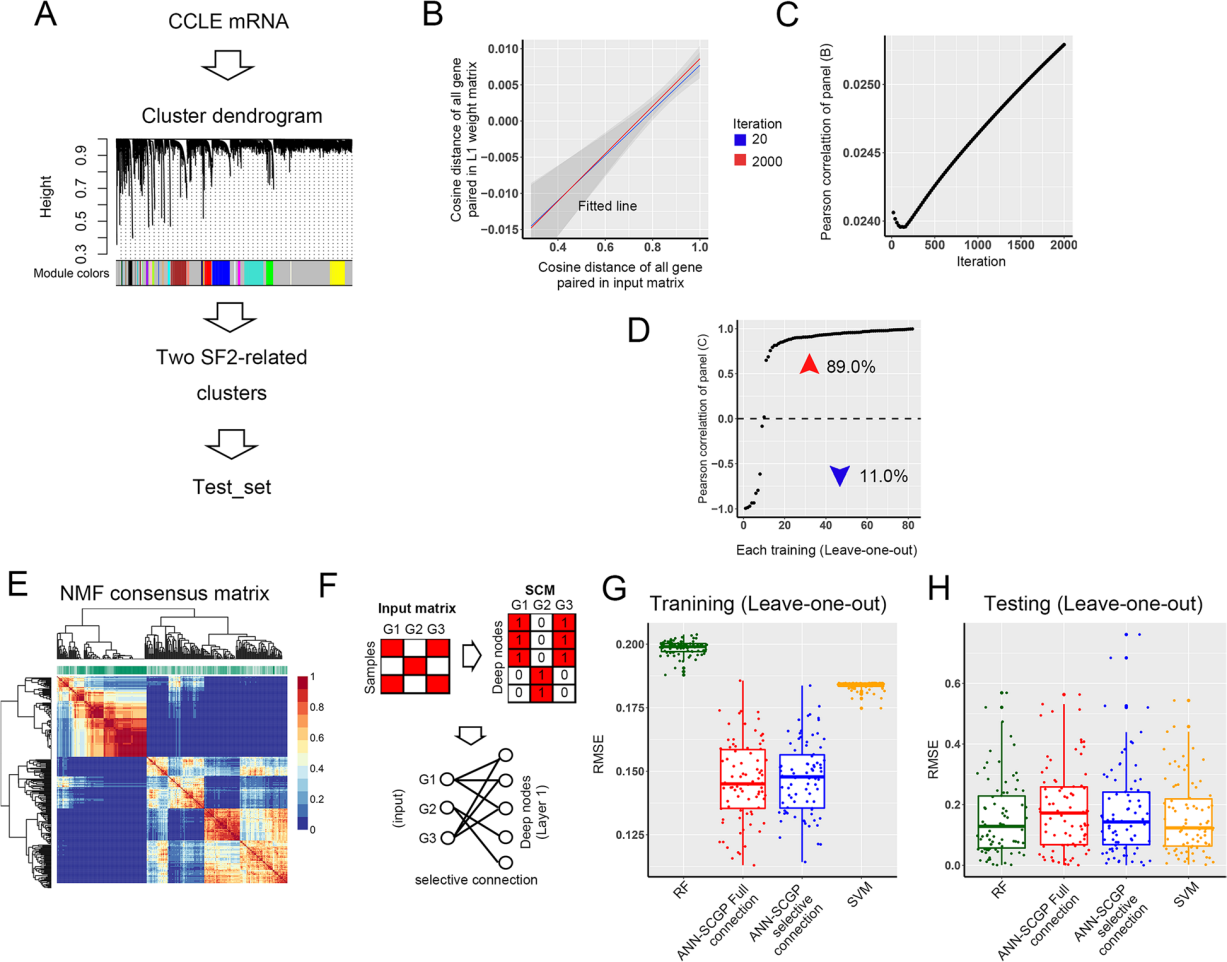
ANN-SCGP model with selective connection showed stronger generalization ability than models with full connection. **A** Constructing Test_set as discovery set using WGCNA. **B** & **C** The correlation of gene similarity of L1 weight matrix and the input matrix increased with it-erations in 1 of the 82 leave-one-out training. **D** The correlation of gene similarity of L1 weight matrix and the input matrix increased with iterations in 73 of the 82 leave-one-out training. **E** & **F** Illustration of constructing SCM via NMF. **G** RMSE of the 4 models in training. **H** RMSE of the 4 models in testing. WGCNA, weighted gene co-expression network analysis; RMSE, root mean squared error; NMF, non-negative matrix factorization; SCM, selectively connected matrix

To compare the selective connection of ANN-SCGP with full connection, we constructed a discovery set (namely Test_set) with 82 samples from CCLE. The input genes were from the 2 SF2-related clusters in weighted gene co-expression network analysis (WGCNA, Fig. 2A) [29]. We next trained ANN-SCGP with full connection and 1 hidden layer (Supplementary Fig. 3) to fit SF2, and used the leave-one-out cross-validation. Interestingly, we observed that the correlation of distance of genes in the L1 weight matrix and the input matrix increased with 2000 iterations (Fig. 2B & C). In 82 times of training using the leave-one-out cross-validation, the above correlations of 89% training were increased with iterations (Fig. 2D), which implied a tendency in models with deeper iterations (smaller errors) that similar genes have similar deep node connection patterns. The above results suggest that SCM mapped by gene similarity may benefit model performance (Fig. 2E & F). We then compared the performance of the 4 models: ANN-SCGP, ANN with full connection, SVM, and RF using the leave-one-out cross-validation method. ANN-SCGP had a satisfactory performance on training and testing (Fig. 2G & H). Compare to ANN-SCGP with full connection, ANN-SCGP showed stronger generalization ability.

### ANN-SCGP accurately predicted SF2

#### Evaluation using the CCLE dataset

For the CCLE dataset of the 82 cell lines, we trained an ANN-SCGP model containing 2 hidden layers, 61 deep nodes, and 1 SF2-predicted regressor on the final layer (Supplementary Fig. 4). We performed fivefold cross-validation in ANN-SCGP, fully-connected ANN, LM, SVM, RF, RSI, PLS, LASSO LM, and elastic net regression to predict SF2. In the training task, LM and PLS showed strong fitting performance, and their RMSE was almost 0 (Tab. 1). The mean RMSE of ANN-SCGP was 0.0943, which was sequentially weaker than LM, PLS, SVM, ANN, and RF, but stronger than regularization-based regression (LASSO LM and elastic net regression). In the testing task, ANN-SCGP outperformed other methods (mean RMSE: 0.1587 in ANN-SCGP; 0.1716 in ANN; 0.1830 in LASSO LM; 0.1834 in PLS; 0.1870 in elastic net regression; 0.1871 in RF; 0.1986 in SVM; 0.4167 in RSI; 1.9304 in LM). The above results suggested that the ANN-based model predicted SF2 better than the traditional machine learning model, and the use of regularization improved the performance of linear regression.

**Table 1:** Comparison of predictive performance of models for SF2 in CCLE and GDSC

	**Methods**	**Training (RMSE)**						**Testing (RMSE)**					
		**Mean**	**CV1**	**CV2**	**CV3**	**CV4**	**CV5**	**Mean**	**CV1**	**CV2**	**CV3**	**CV4**	**CV5**
**CCLE**	ANN-SCGP	0.0943	0.1083	0.0805	0.0757	0.1175	0.0893	**0.1587**	**0.1578**	**0.1733**	0.1750	**0.1435**	0.1441
	ANN	0.0479	0.0681	0.0305	0.0421	0.0501	0.0489	0.1716	0.1671	0.2004	0.1638	0.1621	0.1648
	LM	**0.0000**	**0.0000**	**0.0000**	**0.0000**	**0.0000**	**0.0000**	1.9304	0.8530	0.7878	2.6586	1.1412	4.2116
	SVM	0.0342	0.0208	0.0883	0.0208	0.0209	0.0201	0.1986	0.2155	0.2028	0.1826	0.2042	0.1882
	RF	0.0746	0.0694	0.0734	0.0785	0.0738	0.0781	0.1871	0.2087	0.2161	0.1639	0.1848	0.1619
	RSI	0.4186	0.4200	0.4231	0.4137	0.4336	0.4023	0.4167	0.4132	0.3999	0.4386	0.3559	0.4761
	PLS	**0.0000**	**0.0000**	**0.0000**	**0.0000**	**0.0000**	**0.0000**	0.1834	0.1831	0.2114	0.1790	0.1758	0.1676
	LASSO LM	0.1434	0.1627	0.1406	0.1372	0.1825	0.0942	0.1830	0.2068	0.2261	**0.1580**	0.1889	**0.1355**
	Elastic Net	0.1402	0.1765	0.1389	0.1082	0.1811	0.0962	0.1870	0.2116	0.2272	0.1687	0.1891	0.1385
**GDSC**	ANN-SCGP	0.0993	0.0935	0.1017	0.0852	0.1056	0.1104	**0.1654**	**0.2099**	**0.1845**	**0.1183**	**0.1511**	0.1628
	ANN	0.0433	0.0431	0.0479	0.0394	0.0524	0.0340	0.1826	0.2224	0.1928	0.1684	0.1771	**0.1522**
	LM	**0.0000**	**0.0000**	**0.0000**	**0.0000**	**0.0000**	**0.0000**	28.4256	41.6402	90.0651	0.9031	8.8523	0.6675
	SVM	0.0604	0.0204	0.0864	0.0869	0.0218	0.0865	0.2020	0.2633	0.2042	0.1539	0.1848	0.2040
	RF	0.0790	0.0697	0.0811	0.0830	0.0825	0.0790	0.2049	0.2697	0.2284	0.1662	0.1603	0.1998
	RSI	0.4437	0.4478	0.4535	0.4428	0.4506	0.4240	0.4412	0.4276	0.4026	0.4485	0.4158	0.5115
	PLS	**0.0000**	**0.0000**	**0.0000**	**0.0000**	**0.0000**	**0.0000**	0.2252	0.2457	0.2116	0.2044	0.2300	0.2345
	LASSO LM	0.2014	0.1977	0.2046	0.2191	0.1857	0.1997	0.2089	0.2578	0.2636	0.1598	0.1620	0.2012
	ElasticNet	0.1843	0.1882	0.2046	0.2237	0.1907	0.1143	0.2128	0.2580	0.2636	0.1646	0.1625	0.2154

*ANN* Artificial neural networks, *LM* Linear regression model, *SVM* Support vector machine, *PLS* Partial least squares, *RF* Random forest, *RSI* Radiosensitivity index in Torres-Roca's study, *CV* Cross validation, *RMSE* Root mean squared error

#### Evaluation using the GDSC dataset

For the GDSC dataset of 71 cell lines, we trained an ANN-SCGP model with the same construction as the model in the CCLE dataset. After fivefold cross-validation, in the testing task, the best to worst performing algorithms in order were LM, PLS, ANN, SVM, RF, ANN-SCGP, elastic net regression, LASSO LM, and RSI (Tab.**1**). In the testing task, the mean RMSE of ANN-SCGP was 0.1654, which was sequentially stronger than ANN, SVM, RF, LASSO LM, elastic net regression, PLS, RSI, and LM. The results for the independent dataset GDSC and CCLE were similar.

#### Nodes with high contributions were linked to DNA damage response

**Fig. 3:**
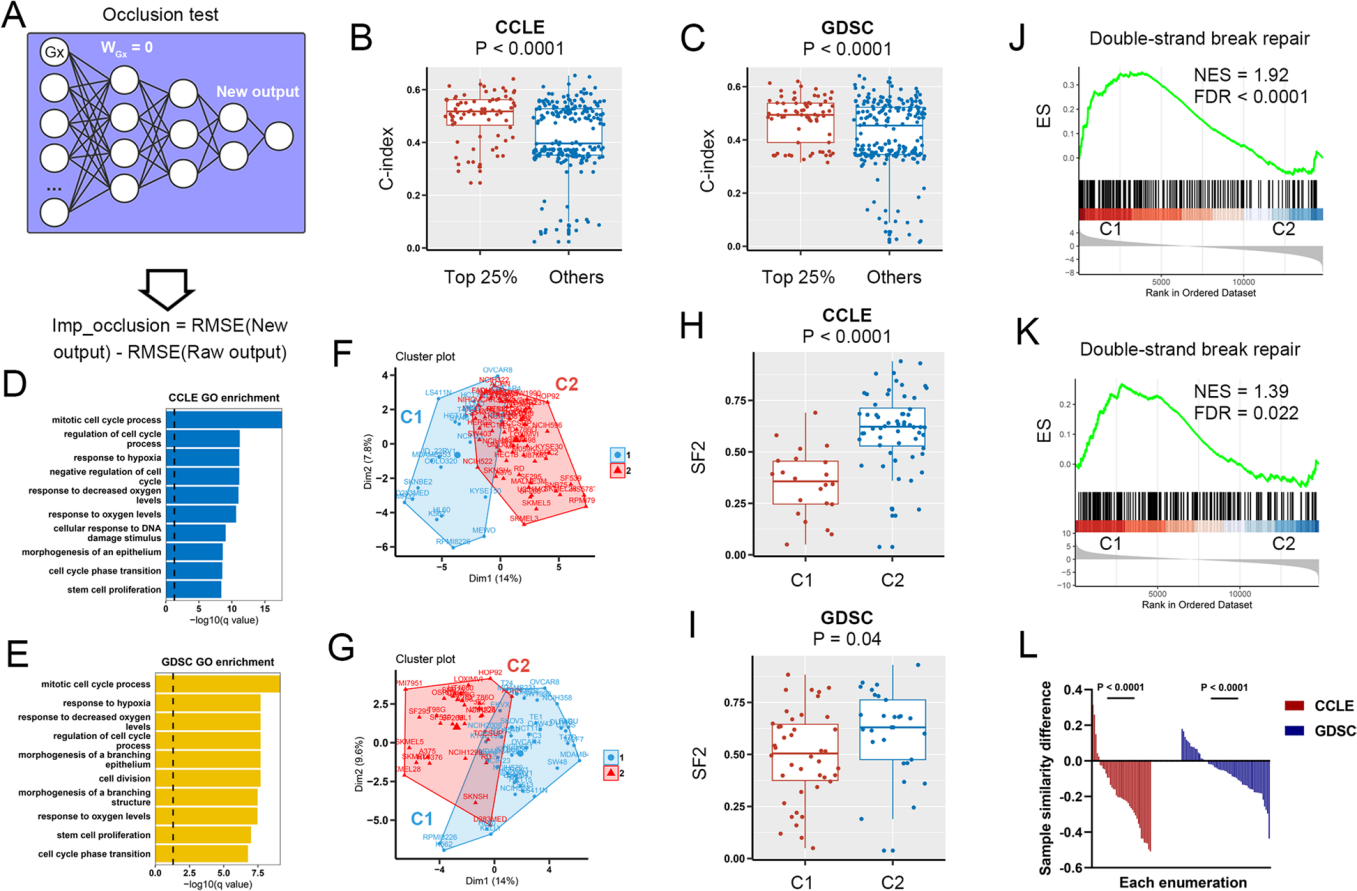
ANN-SCGP accurately predicted SF2. **A** Illustration of occlusion test. The genes with top 25% occlusion scores had significantly higher SF2-predictive C-indexes than others in CCLE **B** and GDSC **C**. Gene ontology analysis was performed by top 25% occlusion score genes in CCLE **D** and GDSC **E**. We clustered samples into two groups (C1 & C2) via K-means of genes with top 25% occlusion scores in CCLE **F** and GDSC **G**. C2 samples had high SF2 in CCLE **H** and GDSC **I**. C1 was linked to high signals of double-strand break repair in CCLE **J** and GDSC **K**. **L** The horizontal coordinate was the enumeration of all combinations of node-based and gene-based sample similarity from different groups (controls). The vertical coordinate was the Pearson's correlation of node-based and gene-based sample similarity from controls minus that from same group. RMSE, root mean squared error; CCLE, Cancer Cell Line Encyclopedia; GDSC, Drug Sensitivity in Cancer; LM, linear regression model; SVM, support vector machine; RF, random forest; RSI, radiosensitivity index

To assess the contributions of genes in ANN-SCGP, we performed the occlusion test (see Sect. 2.2.6) in CCLE and GDSC datasets (Fig. 3A). The genes with the top 25% occlusion scores had significantly higher SF2-predictive C-indexes than others (Fig. 3B & C). Enrichment analysis showed that the top 25% contributed genes were significantly associated with cell cycle, DNA damage stimulus, and response to hypoxia (Fig. 3D & E). According to the top 25% contributed signatures, we clustered samples into 2 groups (C1 & 2) through K-means (Fig. 3F & G). Compared with C2, the C1 samples had lower SF2 in CCLE (t.test *p* < 0.0001, Fig. 3H) and GDSC datasets (t.test *p* = 0.04, Fig. 3I). Gene set enrichment analysis (GSEA) [30] showed that C1 was linked to high signals of double-strand break repair (Fig. 3J & K).

#### Low dimensional features of ANN-SCGP can reproduce gene patterns

Next, we focused on the biological interpretability of ANN-SCGP. Since the selective connection layer was mapped by gene similarity, we were concerned whether the first hidden layer learned the gene similarity pattern. Through consensus clustering of SCM, we clustered the L1 layer low-dimensional nodes of GDSC and CCLE into 5 groups each (Supplementary Fig. 5). In the SCM, genes with weights "on" in more than 50% of the low-dimensional nodes of a group were considered to be group-specific. For a given group, we calculated the node-based sample similarity (value was the Pearson's correlation coefficient) according to the output of the group-specific nodes in the first hidden layer. Similarly, we calculated the gene-based sample similarity according to the group-specific genes at the input. The gene-based sample similarity and node-based sample similarity from different groups were used as controls. Enumerating all combinations of groups in 5 cross-validations, the consistency of node-based and gene-based sample similarity from the same group was observed to be greater than the controls in both CCLE and GDSC (Fig. 3L). The above results confirmed that the hidden layer of the ANN-SCGP model successfully learns the gene pattern features.

#### Epistemic uncertainty of ANN-SCGP in CCLE and GDSC

Deep ensembles method was demonstrated to be excellent and robust for predicting uncertainty [31]. Here, the ensemble-based approach was used to train the entire dataset by different initialization of the weights and obtained a total of 5 models (M = 5, this was similar to Deep Ensembles but without adding additional variance prediction node in the output layer). Next, we calculated the standard deviation of the 5 model output values as epistemic uncertainty [32]. In CCLE, uncertainty values of the sample from 0.0094 to 0.085. We found that the uncertainty value was positively correlated with the RMSE of the mean prediction values of samples in the fivefold cross-validation (Supplementary Fig. 6, *R* = 0.402, *P* = 0.00018). In GDSC, the range of uncertainty was from 0.0058 to 0.748. Similarly, we found a significant correlation between uncertainty and RMSE (Supplementary Fig. 7, *R* = 0.626, *P* < 0.0001).

#### Comparison of SF2 prediction in the dataset from the He's study

For a fair benchmark, we compared the SF2-prediction performance of ANN-SCGP with that of SVM and PLS in the dataset with 60 cell lines from He's study [16]. The input genes were the same as those of the He's study. For the ANN-SCGP, we trained a model containing 2 hidden layers (L1: 20 nodes; L2: 10 nodes) and 1 SF2-predicted regressor. In training of threefold cross validation, PLS had the strongest fitting ability (mean RMSE: 0.0004), followed by SVM (mean RMSE: 0.067) and ANN-SCGP (mean RMSE: 0.0717, Supplementary Table 3). In testing, ANN-SCGP achieved the best performance (mean RMSE: 0.1282).

#### ANN-SCGP accurately predicted radiocurability

We next practiced ANN-SCGP with 2 hidden layers (Supplementary Fig. 8) in 1,036 patients with RT from the 10 TCGA pan-cancer datasets whose proportion of censored patients < 80%: Cervical squamous cell carcinoma and endocervical adenocarcinoma (CESC), Esophageal carcinoma (ESCA), Brain Lower Grade Glioma (LGG), Head and Neck squamous cell carcinoma (HNSC), Lung adenocarcinoma (LUAD), Lung squamous cell carcinoma (LUSC), Pancreatic adenocarcinoma (PAAD), Sarcoma (SARC), Stomach adenocarcinoma (STAD), and Uterine Corpus Endometrial Carcinoma (UCEC). The inclusion and exclusion strategies were shown in supplementary Fig. 9.

The clinical and biological heterogeneity of different tumors was very large (Supplementary Fig. 10A & B). Therefore, we next trained ANN-SCGP, DeepSurv (a fully connected ANN model) [33], RSF, CPH, LASSO CPH, and RSI models in each tumor, respectively. Due to the limited sample sizes, we conducted twofold cross-validation. For TCGA-HNSC with 264 patients, ANN-SCGP significantly predicted the prognosis of patients with radiotherapy (Supplementary Fig. 10C-F). Multivariate Cox regression showed that the predictive value of ANN-SCGP was an independent prognostic factor (Supplementary Fig. 10G-J). For 5-year survival, ANN-SCGP had the highest AUC of ROC in both training and testing (Supplementary Fig. 10 K-N). Similar results were observed in the other 9 TCGA datasets (Table 2, Supplementary Table 4 & 5). Overall, for training, CPH had the strong fitting ability in all datasets (all C-index = 1); ANN-SCGP showed 3/20 of 1st and 13/20 of 2nd C-index in the twofold cross-validation of 10 datasets; ANN showed 1/20 of 1st, 1/20 of 2nd and 3/20 of 3rd C-index. For testing, ANN-SCGP showed 12/20 of 1st and 4/20 of 2nd C-index; ANN showed 4/20 of 1st and 5/20 of 2nd C-index; LASSO CPH showed 3/18 of 1st and 3/18 of 2nd C-index; RSF showed 2/17 of 1st and 4/17 of 2nd C-index; RSI showed 1/20 of 2nd, 4/20 of 3rd, and 3/20 of 4th C-index; CPH showed 3/20 of 2nd, 1/20 of 3rd, and 13/20 of opposite prediction C-index.

**Table 2:** Comparison of predictive performance of models for prognosis in TCGA patients with RT

Cohorts		ANN-SCGP		LASSO CPH		ANN		CPH		RSF		RSI	
		**C-index (CV1)**	**C-index (CV2)**	**C-index (CV1)**	**C-index (CV2)**	**C-index (CV1)**	**C-index (CV2)**	**C-index (CV1)**	**C-index (CV2)**	**C-index (CV1)**	**C-index (CV2)**	**C-index (CV1)**	**C-index (CV2)**
LGG	Training	0.958	0.964	0.869	0.840	0.870	0.903	**1.000**	**1.000**	0.930	0.944	0.675	0.658
	Testing	0.787	**0.815**	0.825	0.794	0.753	0.716	OP	OP	**0.839**	0.798	0.658	0.675
HNSC	Training	0.928	0.937	0.790	0.820	0.770	0.784	**1.000**	**1.000**	0.916	0.934	0.524	0.522
	Testing	0.609	0.576	**0.629**	**0.582**	0.570	0.558	0.571	OP	0.608	0.568	0.522	0.524
CESC	Training	0.976	0.972	NA	0.891	0.920	0.881	**1.000**	**1.000**	0.927	0.961	0.539	0.548
	Testing	0.698	0.623	NA	0.646	**0.802**	**0.658**	OP	OP	0.618	0.626	0.548	0.539
SARC	Training	0.948	0.983	0.919	0.732	0.953	0.905	**1.000**	**1.000**	0.934	0.955	0.711	0.559
	Testing	**0.715**	0.621	OP	0.597	0.651	0.592	OP	0.692	0.682	**0.720**	OP	OP
STAD	Training	0.890	0.981	0.877	0.913	0.881	0.919	**1.000**	**1.000**	0.877	0.944	0.699	0.631
	Testing	**0.906**	**0.730**	0.538	0.558	0.761	0.727	0.631	0.521	0.756	0.656	0.631	0.699
UCEC	Training	1.000	0.957	1.000	0.936	1.000	0.936	**1.000**	**1.000**	NA	NA	0.783	0.532
	Testing	**0.702**	0.826	0.638	0.783	**0.702**	**0.957**	OP	OP	NA	NA	0.532	0.783
ESCA	Training	**1.000**	**1.000**	0.895	0.971	0.947	0.882	**1.000**	**1.000**	0.885	NA	0.842	0.794
	Testing	**0.613**	OP	OP	**0.868**	OP	OP	0.516	0.581	0.516	NA	OP	OP
LUAD	Training	0.990	0.817	0.620	0.875	0.750	0.663	**1.000**	**1.000**	0.923	0.942	0.644	0.548
	Testing	**0.692**	**0.683**	0.577	0.481	0.567	0.548	OP	OP	0.567	OP	0.548	0.644
PAAD	Training	0.964	0.888	0.813	NA	0.855	0.838	**1.000**	**1.000**	0.928	0.900	0.554	0.600
	Testing	**0.663**	**0.639**	OP	NA	0.625	0.596	OP	OP	0.550	OP	0.600	0.554
LUSC	Training	0.982	0.938	0.754	0.979	0.754	0.729	**1.000**	**1.000**	0.930	0.917	0.588	0.563
	Testing	**0.563**	**0.772**	OP	OP	OP	0.667	OP	0.614	0.542	0.526	OP	OP

*ANN* Artificial neural networks, *CPH* Cox proportional hazard model, *RSF* Random survival forest, *RSI* Radiosensitivity index in Torres-Roca's study, *OP* the prediction directions of training and testing values were opposite, *CV* Cross validation, *NA* Not available

To evaluate the prognostic value of high occlusion score signatures under the different cut-off points of occlusion scores, we performed the occlusion test and developed a traversal method in all TCGA datasets (see traversal method in Additional file 1). In all tumors, the genes with high occlusion scores were more predictive of prognosis except SARC (Supplementary Table 6).

#### Immunity and DNA damage responses were associated with radiocurability

Based on the twofold cross-validation ANN-SCGP model, we calculated 2 radiocurability values (output of ANN-SCGP) for each patient in TCGA datasets. Next, we divided each patient into the high and low radiocurability groups (low radiocurability: both the 2 radiocurability values < median; high radiocurability: others), which showed the distinct prognosis (Supplementary Fig. 11).

**Fig. 4:**
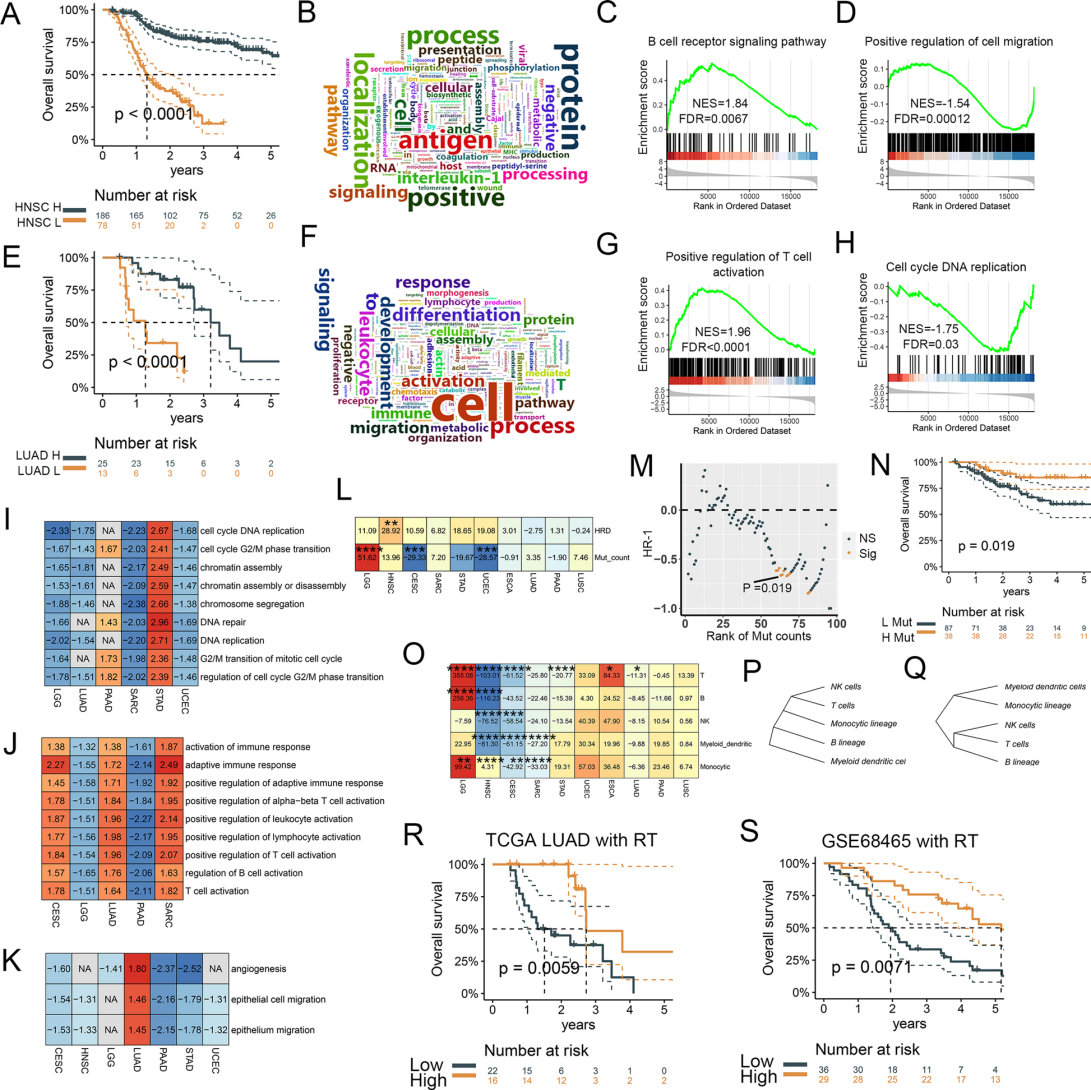
Immunity and DNA damage response were the prognostic factors of radiotherapy. **A** High radiocurability patients showed favorable OS in HNSC. **B** Word clouds of GSEA between high-low radiocurability patients in HNSC. **C** & **D** GSEA between high-low radiocurability patients in HNSC. **E–H** Survival curve, word clouds, and GSEA of LUAD. **I-K** Pan-cancer analysis of GSEA revealed that immunity, DDR, and angiogenesis influenced radiocurability. The value is the normalized enrichment score of GSEA for differentially expressed genes between high-low radiocurability patients. **L** The prognostic value of HRD scores and mutation counts via traversal method (see traversal method in Additional file 1). **M** The cut-off-HR scatters of mutation counts in CESC. **N** Survival curve of high-low mutation counts in CESC. **O** The prognostic value of immune infiltration via traversal method. Hierarchical clustering of SCM **Q** can effectively characterize the similarity of immune infiltration matrix **P**. **R** & **S** ANN-SCGP model performed well in both training (TCGA LUAD) and testing (GSE68465) sets using immune infiltration matrix as inputs. RT, radiotherapy; SCM, selectively connected matrix; Mut, mutation; HR, hazard ratio

For TCGA HNSC, we performed GSEA in high and low radiocurability groups (Fig. 4A), and word clouds of enriched GO terms were linked to immune signals (Fig. 4B). High B cell receptors and low cell migration were enriched in the high radiocurability group (Fig. 4C & D). For TCGA LUAD, patients with high T cell activation and low cell cycle DNA replication signals had a relatively better radiocurability (Fig. 4E-H).

Next, pan-cancer analysis of GSEA between high-low radiocurability patients revealed immunity, DNA damage response (DDR), and angiogenesis influenced radiocurability (Fig. 4I-K). Activated adaptive immunity was enriched in the high radiocurability group in LUAD, SARC, and CESC. Activated lymphocyte and DDR-related signals were linked to unfavorable radiocurability in LGG, which was probably caused by its distinctive tumor microenvironment [34, 35]. Except for PAAD and STAD, DDR was not conducive to radiocurability. Except for LUAD, high radiocurability group had low angiogenesis signals. We next investigated the prognostic values of HRD scores and mutation counts via traversal method (see traversal method in Additional file 1, Supplementary Table 7). High HRD scores were linked to unfavourable OS in HNSC (Fig. 4L). However, there was no significant association between HRD scores and radiocurability in other datasets. Mutation counts were related to worse OS in LGG and better OS in gynecological tumors (CESC and UCEC). The detailed cut-off-hazard ratio scatters in CESC were demonstrated in Fig. 4M & N. Furthermore, we investigated the prognostic values of 5 immune cell infiltration (T, B, NK, DC, and monocyte cells) calculated by MCPcounter via traversal method (Supplementary Table 8). Immune infiltration was a favorable factor in radiocurability in HNSC, CESC, and SARC, but an unfavorable factor in LGG (Fig. 4O). High T cells were also beneficial to prognosis in LUAD and STAD.

To further explore the prognostic values of immune infiltration, we next trained ANN-SCGP with 1 hidden layer (Supplementary Fig. 12) in TCGA LUAD using MCPcounter scores of the above 5 immune cells and tested this model in an external LUAD dataset (GSE68465) with 65 patients received RT [36]. The SCM can effectively characterize the similarity of immune cells and distinguish myeloid and lymphoid immune cells (Fig. 4P & Q). ANN-SCGP model performed well in both training and testing sets (Fig. 4R & S).

### ANN-SCGP training with a priori gene interaction matrix

**Fig. 5:**
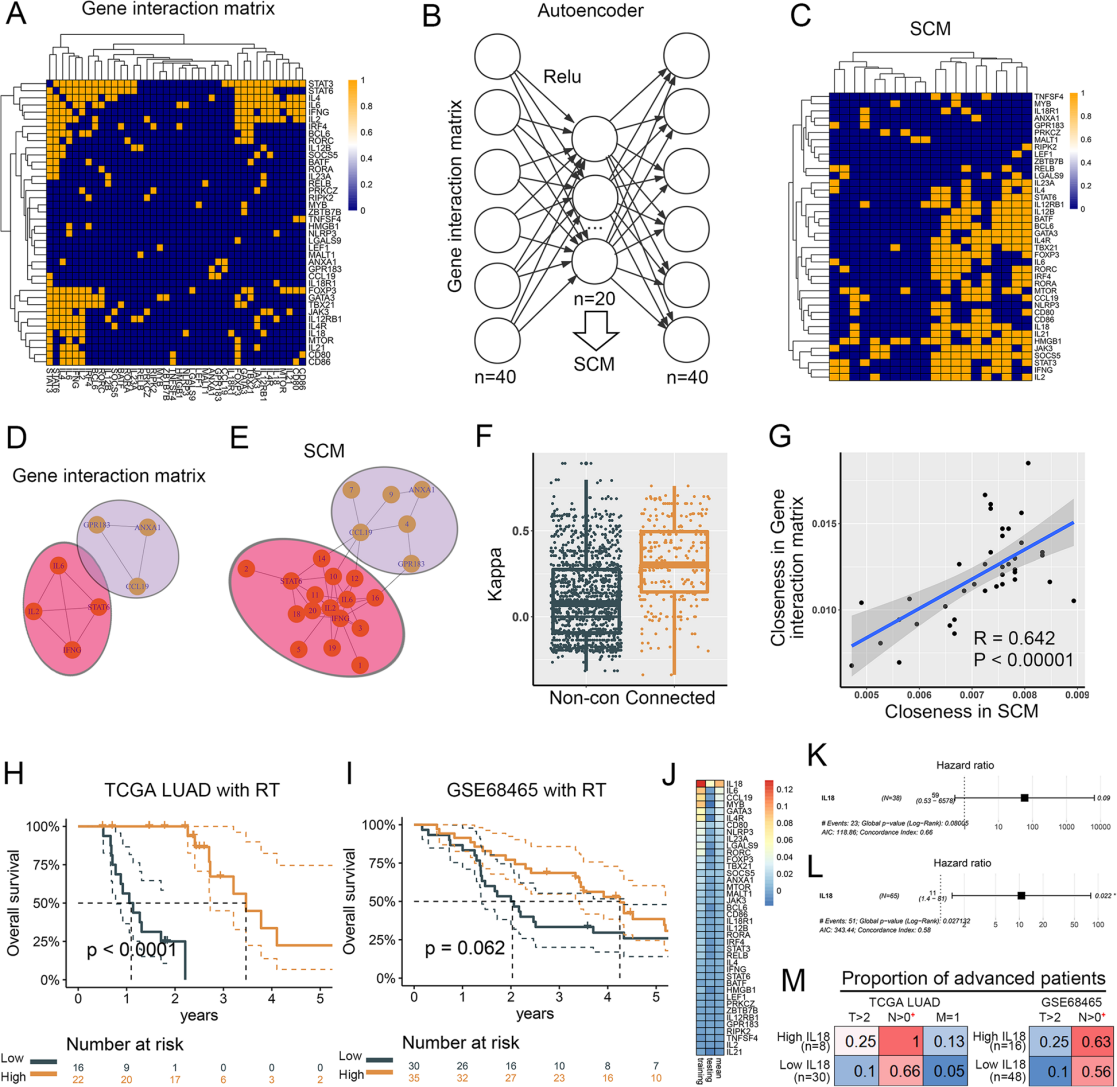
Trained ANN-SCGP using a priori gene interaction matrix. **A** Gene interaction matrix from STRING database. **B** Illustration of autoencoder. **C** SCM identified by autoencoder. **D** & **E** Two connected group genes in gene interaction matrix showed distinct connected patterns in SCM. **F** Interlinked genes had higher Kappa coefficients in SCM than unlinked genes. **G** Nodes of Gene interaction matrix and SCM had similar closeness. **H** & **I** ANN-SCGP model predicted OS in TCGA LUAD (training) and GSE68465 (testing). **J** Occlusion test suggested that IL18 had highest contribution. IL18 was an unfavorable prognostic factor in TCGA LUAD **K** and GSE68465 **L**. **M** High IL18 was linked to advanced patients. RT, radiotherapy; SCM, selectively connected matrix

In the above analysis, we built SCM based on NMF. When there was a priori gene interaction matrix, we trained an autoencoder model to extract SCM (see Sect. 2.2.3) from the deepest hidden layer. We collected T cell activation related genes from GO biological process: alpha beta T cell activation involved in immune response, which had 40 overlaps with TCGA LUAD and GSE68465 datasets. STRING database was used to construct gene interaction matrix (Fig. 5A) with 40 genes and 296 connections whose interaction scores > 0.7 (high confidence, Supplementary Fig. 13) [37]. After 30,000 iterations, SCM well characterized gene interaction matrix patterns (Fig. 5B & C, Supplementary Fig. 14). Two connected gene groups (IL2, IL6, IFNG and STAT6; CCL19, GPR183 and ANXA1) in gene interaction matrix showed distinct connected patterns in SCM (Fig. 5D & E). Interlinked genes had higher Kappa coefficients in SCM than unlinked genes (mean kappa: 0.32 vs. 0.11, *P* < 0.0001, Fig. 5F). Furthermore, gene interaction matrix and SCM had similar topological properties (Fig. 5G).

Next, we performed the ANN-SCGP model with 2 hidden layers using the above SCM in TCGA LUAD (training) and GSE68465 (testing) (Fig. 5H & I). ANN-SCGP outperformed other methods for OS prediction (for training, C-index: 1 in CPH, 0.951 in ANN-SCGP, 0.949 in RSF; for testing, C-index: 0.587 in ANN-SCGP, 0.568 in RSF, reverse in CPH). The occlusion test suggested that IL18 had highest contribution in ANN-SCGP (Fig. 5J). IL18 was an unfavourable prognostic factor (Fig. 5K & L) in both training and testing datasets. Consistent with previous studies, these results were probably due to the pro-invasion and metastatic effect of IL18 (Fig. 5M).

## Discussion

We developed an ANN model with novel architectures to predict radiosensitivity and radiocurability. Compared with traditional ANN, ANN-SCGP had stronger generalization ability and biologically interpretability. In multiple datasets at the cellular and patient level, ANN-SCGP outperformed other traditional machine learning models and normal ANN for SF2 and survival predictions. Considering the lack of practice of gene-based ANN in the field of radiosensitivity, ANN-SCGP could contribute to this field.

The main motivation of this study was to introduce gene pattern information into the ANN model. The main idea was to map gene similarity or gene regulatory network information to L1 weights and control their "on–off" status. Since similar genes were more likely to be involved in the same biological function. The selective connection strategy using ANN-SCGP enabled neurons in the first hidden layer to receive input from similar genes. We demonstrated that this strategy allowed the neurons of the first hidden layer to learn gene pattern information (Fig. 3L). Perhaps this idea can be extended to other fields unrelated to radiosensitivity.

The biological mechanism of radiosensitivity was not well understood, especially at the population level. Occlusion test of ANN-SCGP showed high contributing genes of ANN-SCGP were associated with DDR. Further, this study showed that radiocurability was associated with immunity. Similar findings have been reported in other large population studies. Dai et al. [38] and Grass et al. [39] found that tumors with high radiosensitivity revealed higher expression of interferon-related signaling pathways and immune cell infiltration. Some preclinical studies had found the possible mechanism of the crosstalk between radiotherapy and immunity. Radiation can activate immune cells and remodel the inflammatory microenvironment through the cGAS-STING pathway [40]. Some studies demonstrated the potential of the combination of RT and immunotherapy [41]. On the other hand, radiotherapy was able to cause lymphocyte subset damage in the peripheral blood of patients [42]. In this study, we found that immune signals were favorable factors of radiocurability in LUAD, SARC, and CESC, but unfavorable factors in LGG and PAAD, which might be due to distinct microenvironments. Immune checkpoint inhibitors had limited efficacy in glioma and PAAD [43, 44]. Jang et al. also found that the worst prognostic group had high immune-related signals in LGG with RT [45]. In conclusion, this study indicated that immunity may play an important role in radiosensitivity, which needs to be confirmed by further clinical studies.

One of the major challenges in the field of radiosensitivity was the identification of key gene signatures, which was also a pre-requisite for training models. Previous studies attempted to identify radiation-related genes based on single omics data, particularly using the transcriptome [4, 46]. However, biological processes were regulated by multi-omics. Lewis et al. demonstrated the excellent performance of multi-omics classification [18]. Therefore, one of the aims of this study was to identify the multi-omics gene signatures associated with radiosensitivity. Moreoer, previous studies explored the radiosensitivity-related genes by the generalized linear model [7], which may not fit nonlinear data well. However, we found that SF2 followed a beta distribution (Additional file 1). Beta regression of genes showed better performance than the linear model to fit SF2. Since Beta distribution was a probability distribution, theoretically, the alpha and beta parameters of beta regression may reflect the number of dead and surviving cells at 2 Gy. Overall, this study identified 288 multi-omic radiosensitivity gene signatures by a non-linear approach, which may be useful for further radiotherapy studies.

In recent studies, ANN has been applied in survival data [21]. There were some strategies of survival analysis in ANN model. One of the strategies was to transform the survival data into discrete data. For instance, Biganzoli et al. discretized the survival time at one month interval to train ANN model [47]. The ability of these ANN to predict prognosis was not superior to the CPH model. Another strategy was to train ANN with the NLPL function. Katzman et al. proposed a fully-connected ANN called DeepSurv, which showed better predictive effects than CPH model in clinical data [33]. In this study, ANN-SCGP predicted survival better than DeepSurv and CPH, which may be explained by the introduction of a selective connection layer associated with gene patterns.

There were some limits in our study: 1) Lack of prospective clinical evidence to support our conclusion; 2) The impact of radiotherapy schemes and fractionations on the prognosis of radiotherapy patients was well known. However, due to the difficulty in obtaining detailed data, the effects of radiotherapy schemes and fractionations were not included in the analysis; 3) The hidden hypothesis of ANN-SCGP was that model performance benefited from learning low-dimensional features related to gene patterns. Although ANN-SCGP showed excellent performance, the computational model may benefit from other features (e.g. status of the few driver genes), which needed further investigation.

## Supplementary Information

**Additional file 1. Supplementary Material** Prediction of Radiosensitivity and Radiocurability Using a Novel Supervised Artificial Neural Network.** Supplementary Fig. 1.** Our 3-step workflow for identifying multi-omics RRS.** Supplementary Fig. 2.** Identification of radiation related signatures.** Supplementary Fig. 3.** ANN-SCGP with full connection and 1 hidden layer to fit SF2.** Supplementary Fig. 4.** ANN-SCGP model contained 2 hidden layers, 61 deep nodes and 1 SF2-predicted regressor on the final layer.** Supplementary Fig. 5.** Consensus clustering of SCM of ANN-SCGP in CCLE and GDSC.** Supplementary Fig. 6.** Scatter plot of uncertainty and RMSE in CCLE.** Supplementary Fig. 7.** Scatter plot of uncertainty and RMSE in GDSC.** Supplementary Fig. 8.** ANN-SCGP with 2 hidden layers and 288 RRS inputs.** Supplementary Fig. 9.** The inclusion and exclusion strategies of TCGA cohorts.** Supplementary Fig. 10.** ANN-SCGP accurately predicted radiocurability.** Supplementary Fig. 11.** Survival curves of high and low radiocurability groups in TCGA cohorts.** Supplementary Fig. 12.** ANN-SCGP with 1 hidden layer.** Supplementary Fig. 13.** Priori gene interactions via STRING.** Supplementary Fig. 14.** Gene-deep node interactions in SCM. ** Additional Figure S1.** SF2 conformed to beta distribution.** Supplementary Table 1.** A summary table of previous gene model studies on radiosensitivity.** Supplementary Table 2.** Radiation-related signatures.** Supplementary Table 3.** Comparison of SF2 prediction in the dataset from the He's study.** Supplementary Table 4.** Multivariate Cox regression analysis in TCGA patients with RT in cross validation 1.** Supplementary Table 5.** Multivariate Cox regression analysis in TCGA patients with RT in cross validation 2.** Supplementary Table 6.** AUC of T value of C-index between high-low occlusion score groups in each cut-off point.** Supplementary Table 7.** AUC of T value of HR of HRD & mutation scores in each cut-off point.** Supplementary Table 8.** AUC of T value of HR of immune infiltration scores in each cut-off point.** Additional Table S1.** The 82 CCLE cell lines with complete omics data and SF2 annotations from previous laboratory studies.** Additional Table S2.** Simple ANN-SCGP model with PQC outperformed CPH in training and testing of all the 4 datasets after 3,000 iterations.

## Data Availability

Our ANN-SCGP R package presented in this study is openly available in [GitHub repository] at [https://github.com/ZengZihang/ANNSCGP]. Publicly available datasets were analyzed in this study. This data can be found here: NCBI GEO repository GSE68465; CCLE database [https://portals.broadinstitute.org/ccle/data]; GDSC data-base [https://www.cancerrxgene.org/gdsc1000/GDSC1000_WebResources/Home.html]; The cbi-oportal [http://www.cbioportal.org/]; Xena [https://xena.ucsc.edu]; Firehose [https://gdac.broadinstitute.org/]; Dataset from He's study was able to obtain from [https://www.ncbi.nlm.nih.gov/pmc/articles/PMC7225787/]; The radiation annotations were obtained via R TCGAbiolinks package.

## Abbreviations

ANN: Artificial neural network
ANN-SCGP: ANN with Selective Connection Based on Gene Patterns
AUC: Area under curve
CCLE: Cancer Cell Line Encyclopedia
CESC: Cervical squamous cell carcinoma and endocervical adenocarcinoma
CNA: Copy number alteration
CPH: Cox proportional hazards
DDR: DNA damage response
ESCA: Esophageal carcinoma
EXP: Gene expression
GDSC: Drug Sensitivity in Cancer
GSEA: Gene set enrichment analysis
HNSC: Head and Neck squamous cell carcinoma
HRD: Homologous recombination deficiency
LGG: Brain Lower Grade Glioma
LM: Linear regression model
LUAD: Lung adenocarcinoma
LUSC: Lung squamous cell carcinoma
Meth: Methylation
Mut: Mutation
NLPL: Negative log partial likelihood
NMF: Non-negative matrix factorization
OS: Overall survival
PAAD: Pancreatic adenocarcinoma
PQC: Partial quadratic cost
RF: Random forest
RMSE: Root mean squared error
ROC: Receiver operating curve
RRS: Radiation-related signatures
RSF: Random survival forest
RSI: Radiosensitivity index
RT: Radiotherapy
SARC: Sarcoma
SCM: Selectively connected matrix
SF2: Survival fraction at 2 Gy
STAD: Stomach adenocarcinoma
SVM: Support vector machine
TCGA: The Cancer Genome Atlas
UCEC: Uterine Corpus Endometrial Carcinoma
