# Supplementary material for: Prediction of radiosensitivity and radiocurability using a novel supervised artificial neural network
Source: BMC Cancer. 2022 Dec 1;22:1243. doi: 10.1186/s12885-022-10339-3 (PMC9713966; doi:10.1186/s12885-022-10339-3)
Supplement: Supplementary file 1 — Additional file 1. Supplementary Material Prediction of Radiosensitivity and Radiocurability Using a Novel Supervised Artificial Neural Network. Supplementary Fig. 1. Our 3-step workflow for identifying multi-omics RRS. Supplementary Fig. 2. Identification of radiation related signatures. Supplementary Fig. 3. ANN-SCGP with full connection and 1 hidden layer to fit SF2. Supplementary Fig. 4. ANN-SCGP model contained 2 hidden layers, 61 deep nodes and 1 SF2-predicted regressor on the final layer. Supplementary Fig. 5. Consensus clustering of SCM of ANN-SCGP in CCLE and GDSC. Supplementary Fig. 6. Scatter plot of uncertainty and RMSE in CCLE. Supplementary Fig. 7. Scatter plot of uncertainty and RMSE in GDSC. Supplementary Fig. 8. ANN-SCGP with 2 hidden layers and 288 RRS inputs. Supplementary Fig. 9. The inclusion and exclusion strategies of TCGA cohorts. Supplementary Fig. 10. ANN-SCGP accurately predicted radiocurability. Supplementary Fig. 11. Survival curves of high and low radiocurability groups in TCGA cohorts. Supplementary Fig. 12. ANN-SCGP with 1 hidden layer. Supplementary Fig. 13. Priori gene interactions via STRING. Supplementary Fig. 14. Gene-deep node interactions in SCM. Additional Figure S1. SF2 conformed to beta distribution. Supplementary Table 1. A summary table of previous gene model studies on radiosensitivity. Supplementary Table 2. Radiation-related signatures. Supplementary Table 3. Comparison of SF2 prediction in the dataset from the He's study. Supplementary Table 4. Multivariate Cox regression analysis in TCGA patients with RT in cross validation 1. Supplementary Table 5. Multivariate Cox regression analysis in TCGA patients with RT in cross validation 2. Supplementary Table 6. AUC of T value of C-index between high-low occlusion score groups in each cut-off point. Supplementary Table 7. AUC of T value of HR of HRD & mutation scores in each cut-off point. Supplementary Table 8. AUC of T value of HR of immune infiltration scores in each cut- [file 12885_2022_10339_MOESM1_ESM.docx]

**Supplementary Material**

Prediction of Radiosensitivity and Radiocurability Using a Novel Supervised Artificial Neural Network

**1. Supplementary Method**

**1.1. Data collection and standardization**

**1.1.1. Cancer Cell Line Encyclopedia (CCLE) and Genomics of Drug Sensitivity in Cancer (GDSC) datasets**

The RNA expression (Exp), methylation (Meth), copy number alteration (CNA), mutation (Mut), and annotation data of cell line samples were collected from CCLE and GDSC databases [[1](#_ENREF_1), [2](#_ENREF_2)]. For Exp, the CCLE RNA-seq data was standardized using Transcripts Per Million and normalized by log2(x+1) [[3](#_ENREF_3)]. Robust Multi-array Average was used for preprocessing RNA arrays in GDSC [[4](#_ENREF_4)]. For Meth, methylation of CpG islands in CCLE and GDSC were measured by reduced representation bisulfite sequencing and illumina Human Methylation 450 BeadChip [[5](#_ENREF_5)]. CNA data were measured by Affymetrix SNP 6.0 arrays. The Mut data were gene-level binary matrices (0 is non- nonsynonymous mutation; 1 is nonsynonymous mutation).

To obtain SF2 values of each cell line, we conducted a comprehensive search on PubMed. The SF2 values of 82 cell lines in CCLE and 71 cell lines in GDSC were accessed from previous laboratory experiments based on cloning formation analysis (**Additional Table S1**) [[6-73](#_ENREF_6)].

**1.1.2. The Cancer Genome Atlas (TCGA) pan-cancer cohorts**

We included 1,036 patients with external RT of primary tumor field or regional site, who had complete omics & overall survival (OS) data in TCGA datasets. The clinical annotations of TCGA Pan-Cancer cohorts were downloaded from cbioportal [[74](#_ENREF_74)]. The radiation annotations were obtained via R TCGAbiolinks package [[75](#_ENREF_75)]. The omics data were downloaded from Xena [[76](#_ENREF_76)] and Firehose [[77](#_ENREF_77)]. The RNA-seq data were normalized by Transcripts Per Million and log2(x+1). The CNA data were estimated by Affymetrix SNP 6.0 array [[78](#_ENREF_78)]. The somatic non-silent mutations were called by Mutect2 [[79](#_ENREF_79)]. The DNA methylation were quantified by Illumina Human Methylation 450K BeadChip [[5](#_ENREF_5)]. The average value of multi-probes matched to the same gene represented single gene methylation. The OS of RT patients was considered as radiocurability.

**1.1.3. Independent prognostic datasets for cost function comparison**

To compare different cost functions, we collected the 4 datasets with survival annotation from Katzman's study [[80-84](#_ENREF_80)], including the WHAS with 1,638 patients, the SUPPORT with 8,873 patients, the METABRIC with 1,904 patients, and the GBSG with 2,232 patients.

(<https://github.com/jaredleekatzman/DeepSurv/tree/master/experiments/data>).

**1.1.4. GSE68465 data**

Transcriptomic data set GSE68465 was accessed from the GEO database [[85](#_ENREF_85)]. We included 65 LUAD patients who received RT. The expression profiling was normalized by MAS5 [[86](#_ENREF_86)].

**1.2. Three-step method to identify RRS**

We developed a 3-step method to identify RRS, which were considered as input features of ANN-SCGP. Due to the close association between tumor histological subtypes & origin organs (HOs) and radiosensitivity, the effects of HOs were required to be corrected as the covariate. In the first step, we attempted to identify radiosensitivity-related signatures (which consisted of signatures associated with SF2 when adjusting HOs, and highly expressed in the HOs significantly linked to high or low SF2) using CCLE datasets with 82 samples. Since HOs were the multiclass variables with few samples in each class, it is not suitable for dummy variable transformation. We first compared SF2 of each HO with others via wlicox.test. We next assigned discretized values to HOs based on wlicox.test (0 was no significance), therefore HOs were transformed into the grade variables (**Supplementary Fig. 2A & B**). Since SF2 conformed to beta distribution (**Additional Figure S1**), multivariate beta regression [[87](#_ENREF_87)] (included each gene and HOs) was trained to investigate SF2-related Exp, Meth, CNA, and Mut, A total of 3,475 signatures (1,226 Exp, 373 Meth, 1857 CNA, and 19 Mut, namely gene set 1) had statistical significance in multivariate beta regression. Compare to the multivariate linear model, the beta model showed lower AIC, BIC, RMSE, higher R2, and C-index of significant genes (**Supplementary Fig. 2C-E**), demonstrating that beta regression could advantageously fit SF2. Next, we performed sample set enrichment analysis (SSEA, **Supplementary Fig. 2F**) to identify 5,108 signatures (2,165 Exp, 1,214 Meth, 1,340 CNA, and 389 Mut, namely gene set 2) highly expressed in the SF2-related HOs. SSEA can effectively recognize the specific genes of each HO (**Supplementary Fig. 2G**). We integrated the gene sets 1 & 2 as cellular radiosensitivity-related signatures.

In the second step, we identified radiocurability-related signatures from 1,477 patients with RT in the 12 TCGA tumors: BRCA, CESC, ESCA, GBM, LGG, HNSC, LUAD, LUSC, PAAD, SARC, STAD, UCEC. The prognostic concordance index (C-index) of each gene was next evaluated in TCGA patients with external irradiation of the primary tumor field or regional site (**Supplementary Fig. 2H**). After excluding tumors with small sample sizes, we included 1,477 patients from 12 tumor types. We then calculated the weighted sum of prognostic C-index based on tumor sample sizes and selected top 25% of step 1 genes (767 Exp, 263 Meth, 751 CNA, and 102 Mut) as radiocurability-related signatures.

Finally, we optimized the gene set (intersection of radiosensitivity- and radiocurability-related signatures) using gene ontology (GO) terms [[88](#_ENREF_88)]. According to 4 Rs in radiobiology [[89](#_ENREF_89)]. The 4Rs-related signatures (**Supplementary Fig. 2I**) were collected from 5 GO terms: DNA repair (GO:0006281), cell cycle (GO:0007049), regulation of angiogenesis (GO:0045765), response to hypoxia (GO:0001666), and stem cell proliferation (GO:0072089). The final RRS were considered the intersection of signatures of the above 3 steps (**Supplementary Table 1, Supplementary Fig. 2J**).

**1.3. Sample set enrichment analysis (SSEA)**

We developed SSEA to identify genes highly expressed in one of the multi-groups. We first constructed sample sets consisted samples of each group. We then performed enrichment analysis for each gene to calculate enrichment score of sample sets. SSEA was realized by GSEA function of clusterProfiler [[90](#_ENREF_90)].

**1.4. Traversal method: evaluation of the predictive values of different cut-off points for continuous variables**

Due to unstable results of different cut-off values, it was not comprehensive to select only one cut-off value of continuous variables. Specifically, we traversed each cut-off value and divided the samples into high-low groups. Next, we compared high-low groups to get the effect values (hazard ratio, HR or T value of T.test of C-index) and P values. The AUC per rank of the effect values was considered as comprehensive evaluation of the predictive values.

For instance, we calculated the C-index of each high occlusion score gene and low score gene for OS at each threshold, and performed T-test for C-index between high occlusion score and low score genes at each threshold. The AUC of the threshold-T value curve was used as a comprehensive measure of the prognostic significance of the occlusion scores. Negative AUC indicated that the genes with high occlusion scores were more predictive of prognosis.

In section 3.5, we calculated the HR values for OS between high-low immune/DDR-related score patients at each threshold. Next, the AUC of the threshold-(HR-1) value curve was used as a comprehensive measure of the prognostic significance of immune/DDR-related scores. Negative AUC indicated that the immune/DDR-related score was linked to better prognosis.

**1.5. Other algorithms used for comparison**

We used linear regression model (LM), LASSO regression, Elastic net regression, support vector machine (SVM), Cox proportional hazard (CPH) model, random forest (RF), random survival forest (RSF), RSI, and fully-connected ANN algorithms to compare with ANN-SCGP in our study. The LM and CPH were realized by R lm and cph functions [[91](#_ENREF_91), [92](#_ENREF_92)]. SVM was a supervised algorithm to solve maximum-margin hyperplane. The SVM was realized by R e1071 package with eps-regression and linear kernel [[93](#_ENREF_93)]. The other parameters of the SVM used the default parameters of the R svm function. In section 3.4, we used radial kernel consistent with previous studies. The RF and RSF were the regressors with multiple decision trees. We used R randomForest [[94](#_ENREF_94)] and randomForestSRC [[95](#_ENREF_95)] packages to train RF, RSF with default settings. The RSI from Torres-Roca's study were linear regression of 10 genes with rank normalization [[96](#_ENREF_96)]. LASSO regression and Elastic net regression were realized by R glmnet package (https://cran.r-project.org/web/packages/glmnet/index.html). The alpha value in the LASSO regression was 1, while the alpha value in the Elastic net regression was 0.5. The lambda value was the ' lambda.min' value of the cv.glmnet function. PLS model were realized by R pls package (https://cran.r-project.org/web/packages/pls/index.html). In SF2 prediction task, we trained fully-connected ANN by ANN-SCGP without SCM. In survival prediction task, we trained Deepsurv method by R survivalmodels package (https://cran.r-project.org/web/packages/survivalmodels/index.html).

**1.6. Other machine learning algorithms**

NMF was the unsupervised algorithm to reduce a high-dimensional matrix to a low dimensional matrix [[97](#_ENREF_97)]. In this study, we trained NMF using brunet method with 100 iterations. K-means clustering was an unsupervised algorithm using the nearest neighbor information to label categories. K-means was realized by R kmeans function [[98](#_ENREF_98)].

**1.7. Other bioinformatics algorithms**

Gene differential expression analysis was used to identify differentially expressed genes in the whole transcriptome of 2 groups through limma package [[99](#_ENREF_99)]. We constructed pre-ordered list using T values of limma output. The GSEA was performed by R clusterProfiler packages for GO (Gene Ontology) corpus [[90](#_ENREF_90)]. WGCNA was used to identify SF2-related gene clusters via R WGCNA package [[100](#_ENREF_100)]. STRING was a search tool for the retrieval of interacting genes/proteins which were used to construct gene interaction matrix [[101](#_ENREF_101)]. We retained only edges with high confidence (interaction scores > 0.7). The closeness centrality of nodes in the gene interaction network was calculated by R igraph package (https://igraph.org/). dbCRSR was a literature-based database included radiosensitivity regulated factors (395 coding genes, 119 non coding RNAs, and 306 compounds) [[102](#_ENREF_102)].

**2. Supplementary Results**

**2.1. Partial quadratic cost function (PQC) was more efficient than the negative log partial likelihood (NLPL) function**

For the censored time-event data, the classical method (e.g. CPH) was to minimize NLPL function [[103](#_ENREF_103)], which had largely computational consumption. Here, we developed a novel cost function named PQC for prognostic training (see Section 2.2.5). To evaluate the performances of NLPL and PQC, we constructed the simple ANN-SCGP model with no hidden layer, one linear regressor, and full connection that was equivalent to the linear regression model. For 10 iterations, ANN-SCGP model with PQC was about 10 times faster than that with NLPL in the 4 independent prognostic datasets [[80-84](#_ENREF_80)]: Worcester Heart Attack Study (WHAS, PQC vs. NLPL: 1.09s vs. 6.88s), the Study to Understand Prognoses Preferences Outcomes and Risks of Treatment (SUPPORT, PQC vs. NLPL: 2.87s vs. 172.44s), the Molecular Taxonomy of Breast Cancer International Consortium (METABRIC, PQC vs. NLPL: 1.03s vs. 10.88s) and the German Breast Cancer Study Group (GBSG, PQC vs. NLPL: 1.14s vs. 9.75s). Furthermore, the simple ANN-SCGP model with PQC performed similarly to CPH in both training and testing on all 4 datasets after 3000 iterations (**Supplementary Table 2**). In conclusion, PQC is a lighter method without compromising accuracy.

**3. Supplementary Figures**


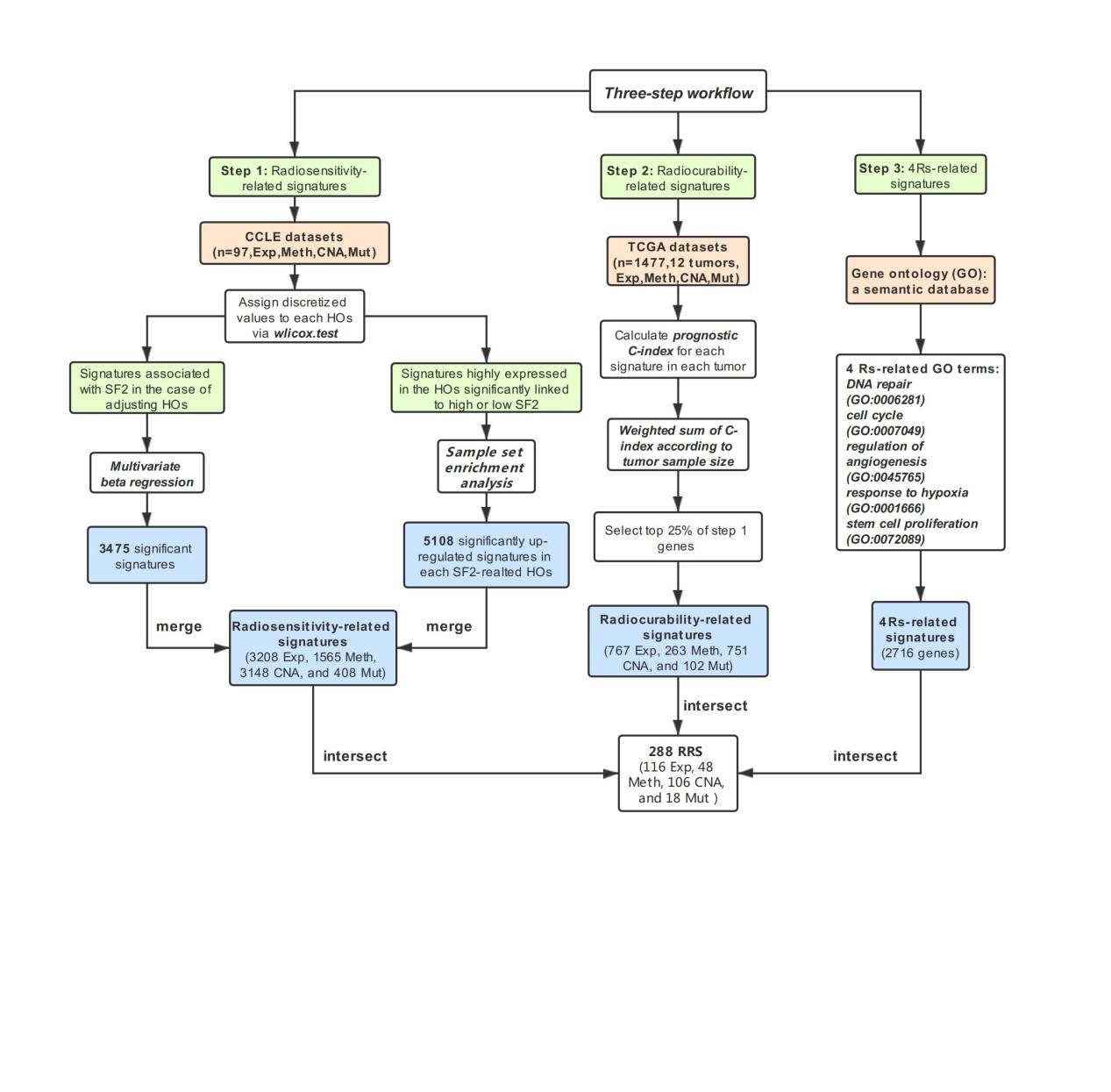


**Supplementary Fig. 1. Our 3-step workflow for identifying multi-omics RRS.** RRS, radiation-related signatures.


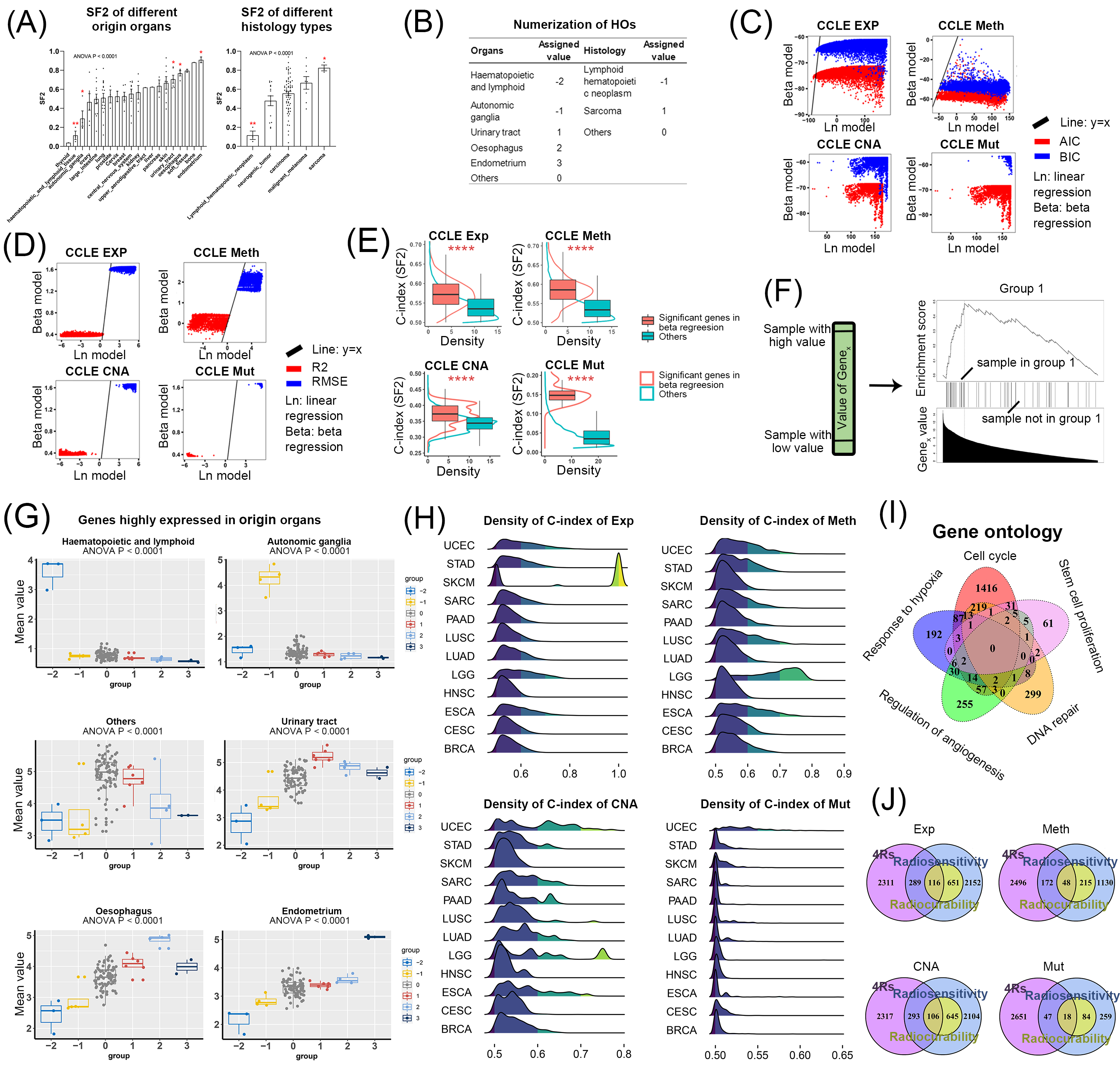


**Supplementary Fig. 2. Identification of radiation related signatures.** (A) SF2 levels in different tumor HOs. (B) Discretized values of HOs. (C) Beta model showed lower AIC and BIC than linear model. (D) Beta model showed lower RMSE and higher R2 than linear model. (E) Significant genes in beta model had higher SF2-predictive C-index than linear model. (F) Illustration of SSEA. (G) SSEA can effectively recognize the specific genes of each HO. (H) Density of prognostic C-index of genes in each TCGA tumor. (I) Genes of 4 Rs-related GO terms. (J) Venn plot of identifying RRS.


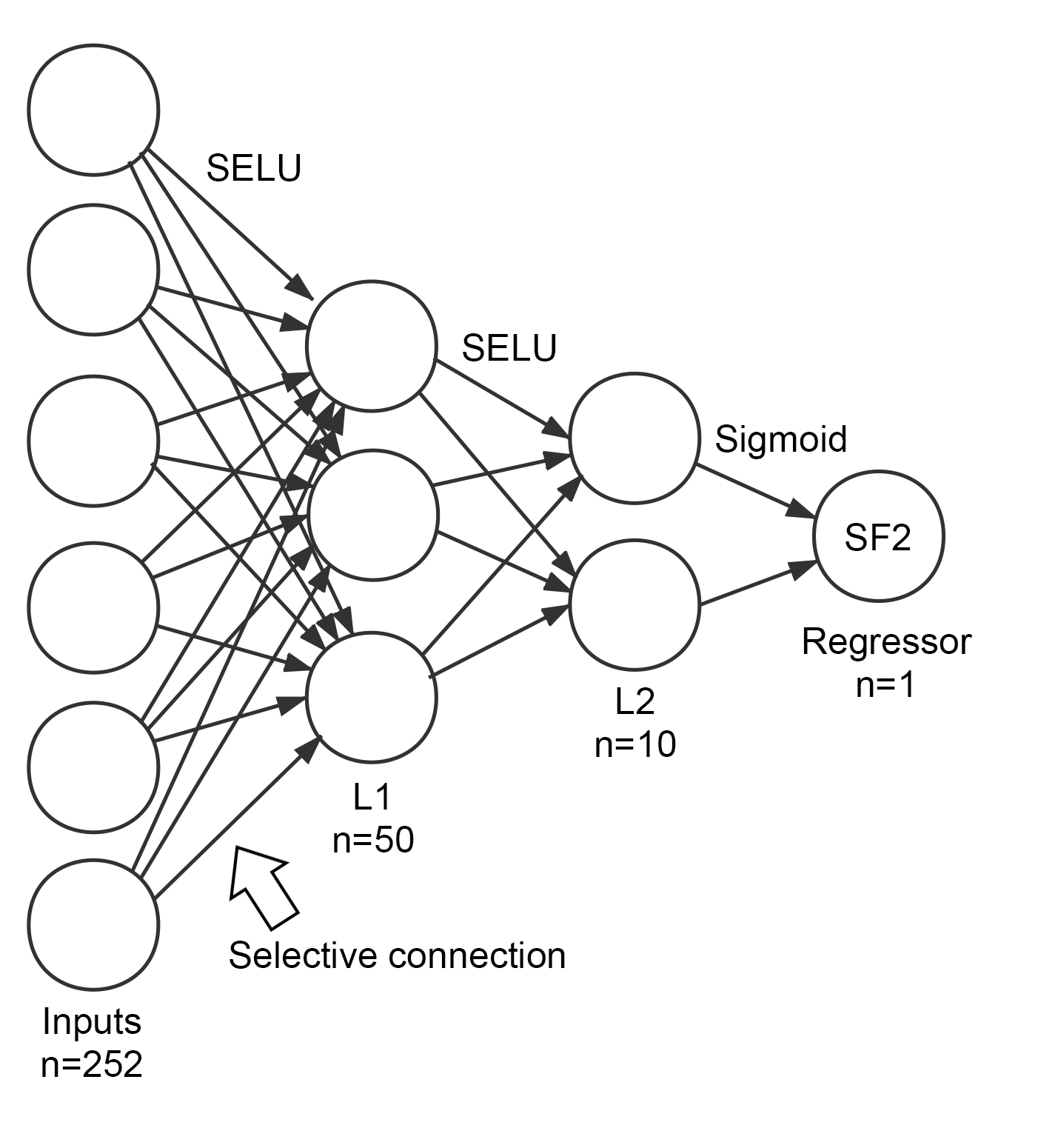


**Supplementary Fig. 3. ANN-SCGP with full connection and 1 hidden layer to fit SF2.**


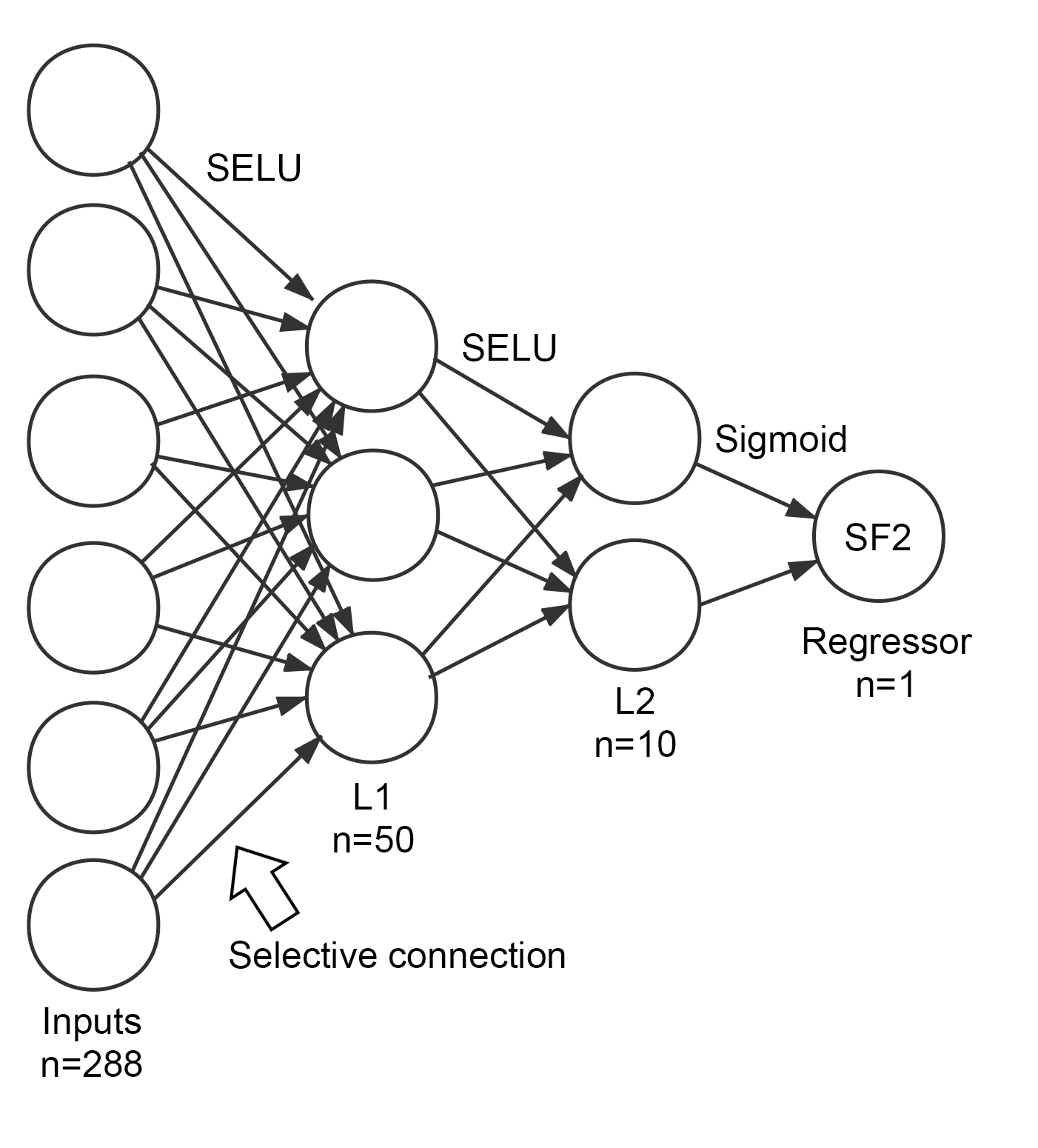


**Supplementary Fig. 4. ANN-SCGP model contained 2 hidden layers, 61 deep nodes and 1 SF2-predicted regressor on the final layer.**

**
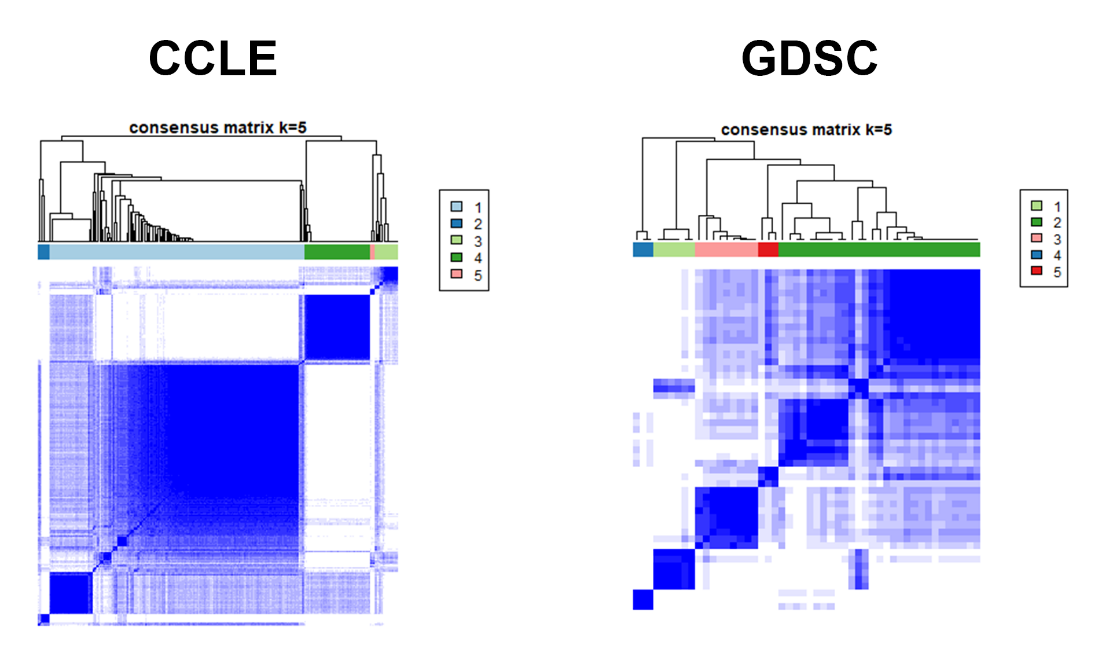
**

**Supplementary Fig. 5. Consensus clustering of SCM of ANN-SCGP in CCLE and GDSC**


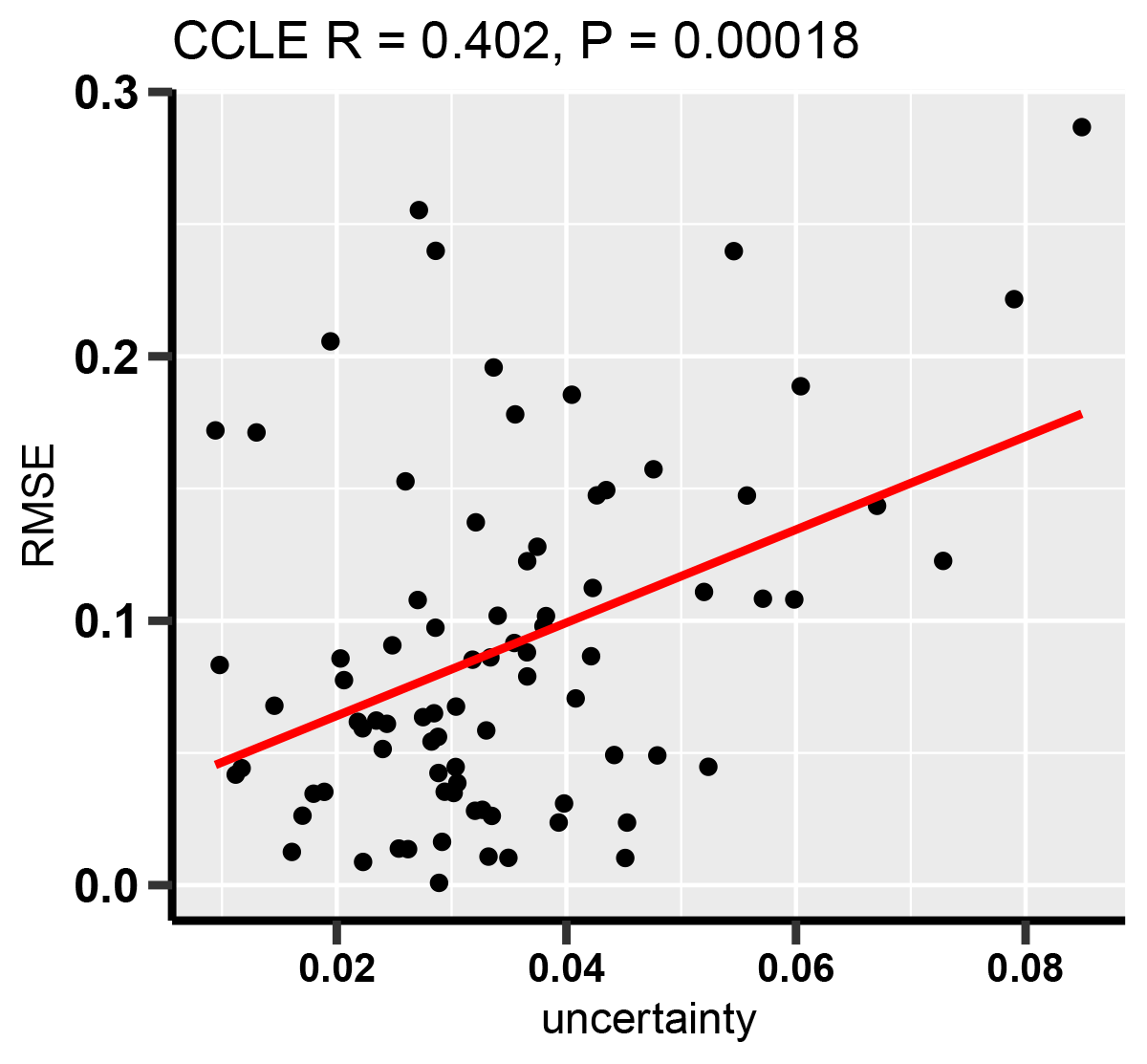


**Supplementary Fig. 6. Scatter plot of uncertainty and RMSE in CCLE.**


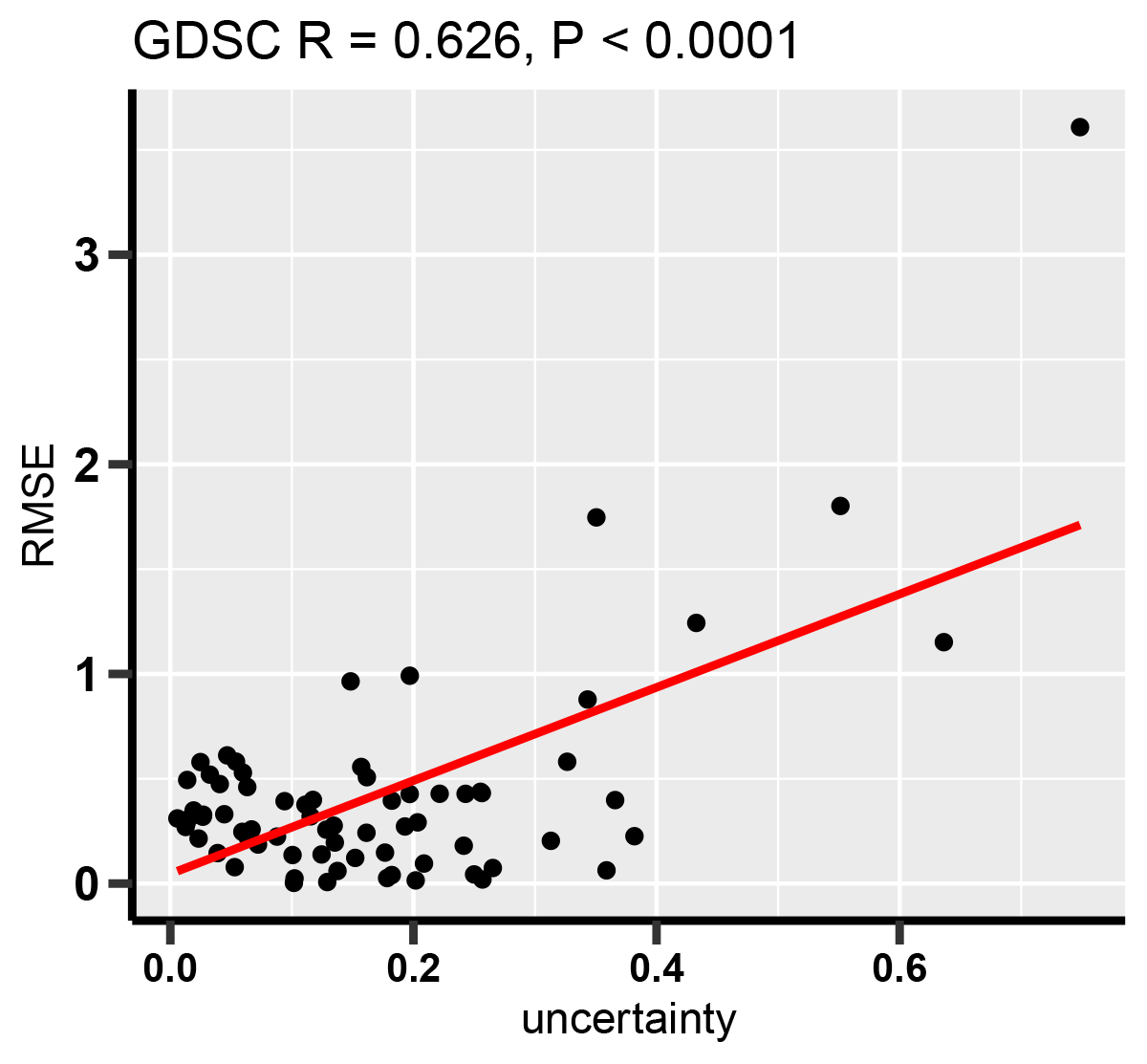


**Supplementary Fig. 7. Scatter plot of uncertainty and RMSE in GDSC.**


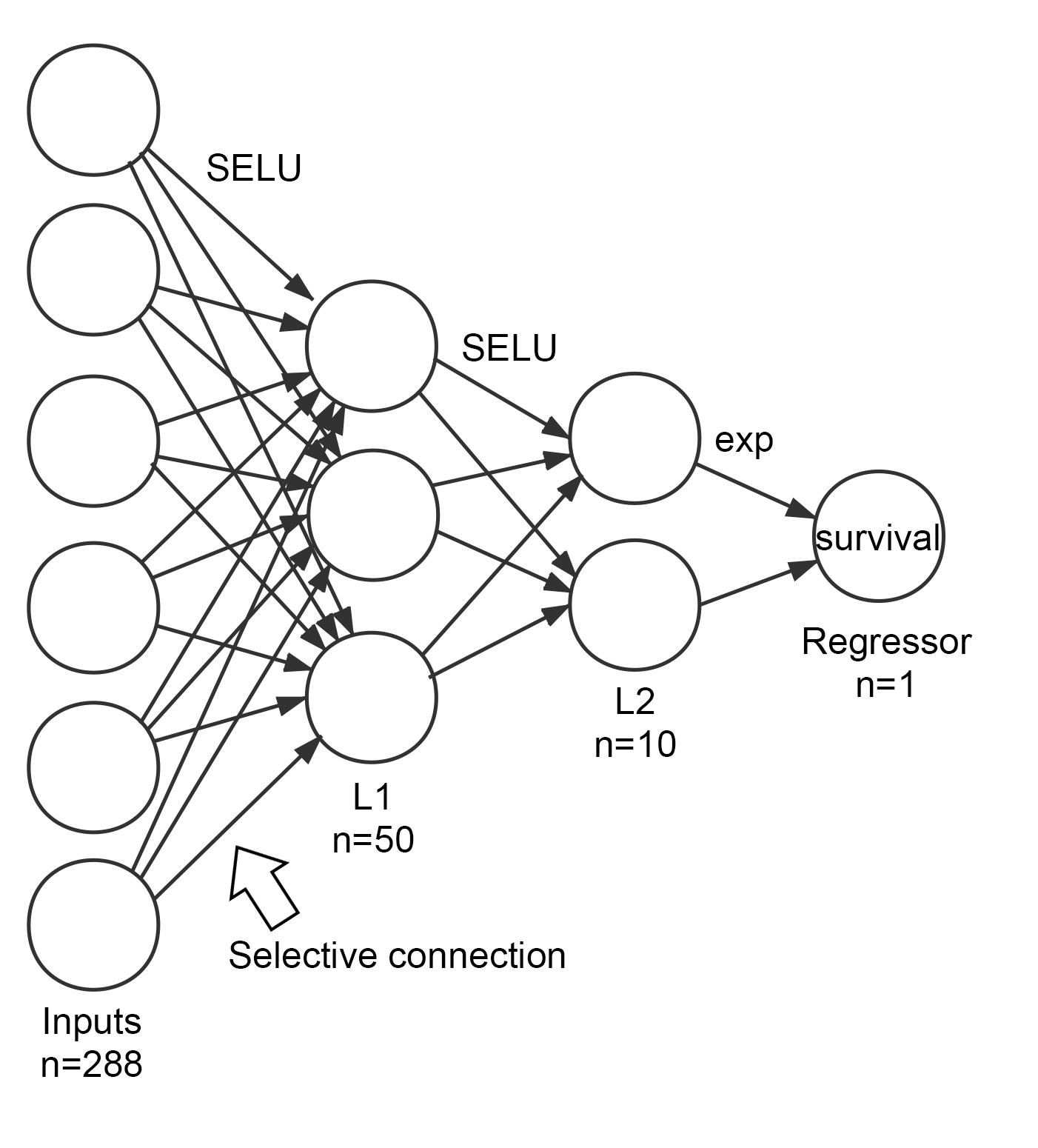


**Supplementary Fig. 8. ANN-SCGP with 2 hidden layers and 288 RRS inputs.**

**
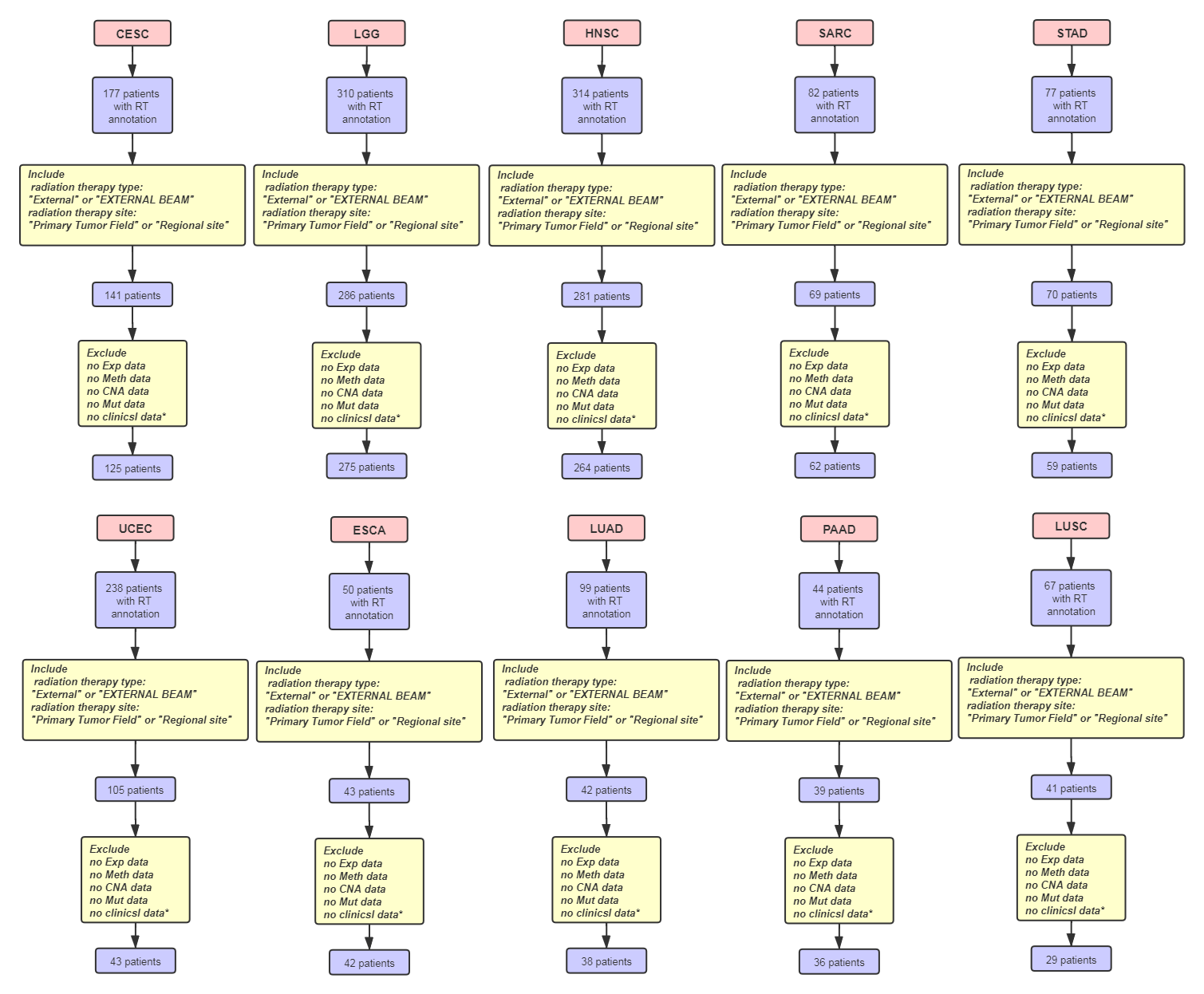
**

**Supplementary Fig. 9. The inclusion and exclusion strategies of TCGA cohorts.**


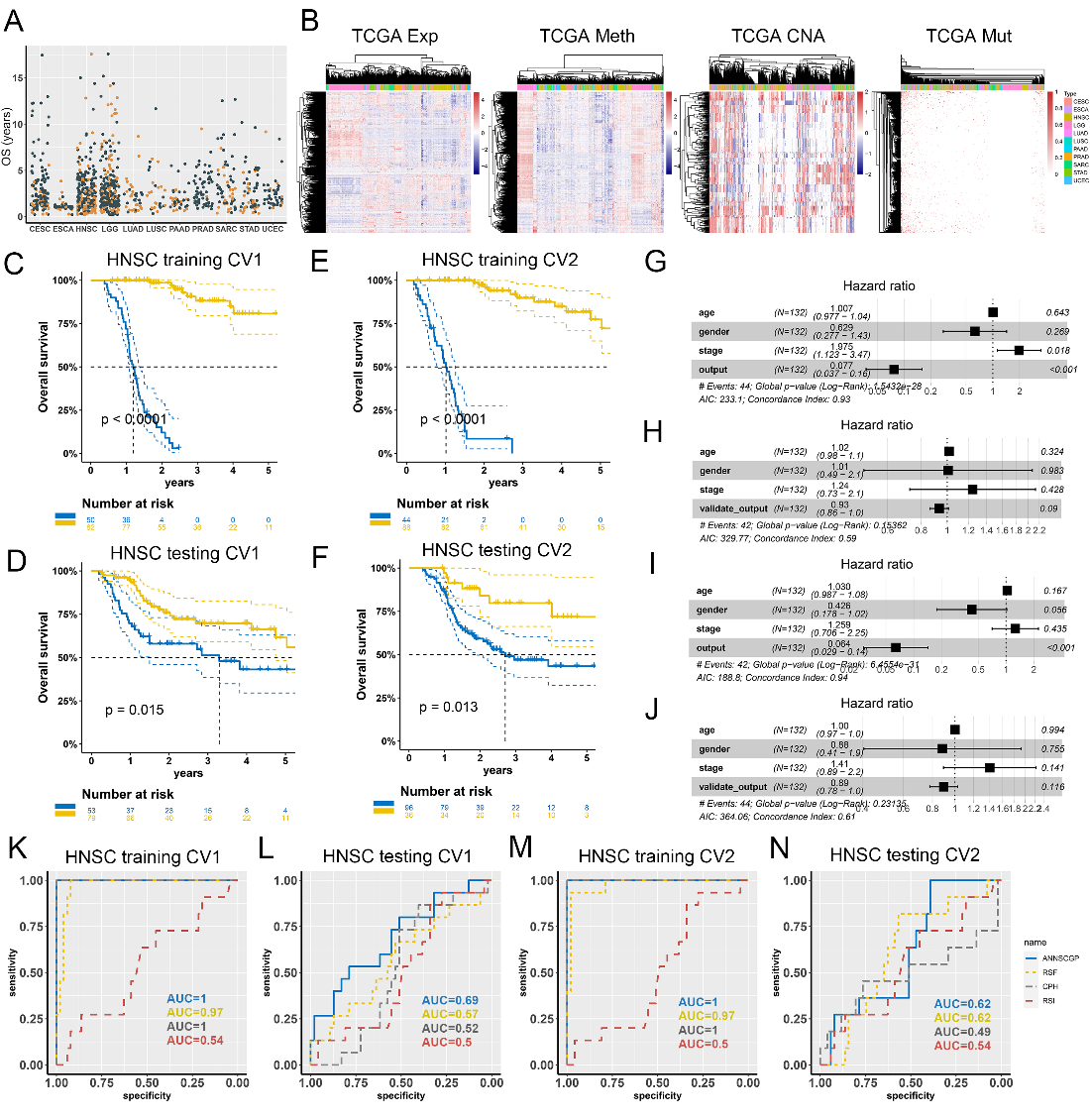


**Supplementary Fig. 10. ANN-SCGP accurately predicted radiocurability.** (A) Prognostic heterogeneity of different tumors in TCGA. (B) Omics heterogeneity of different tumors in TCGA. (C-F) ANN-SCGP can predict OS in HNSC. (G-J) Multivariate Cox regression in HNSC training and testing. (K-N) ANN-SCGP had the highest AUC of ROC of 5-year OS. EXP, gene expression; Meth, methylation; CNA, copy number alteration; Mut, mutation; CV1, cross-validation 1; CV2, cross-validation 2.

**
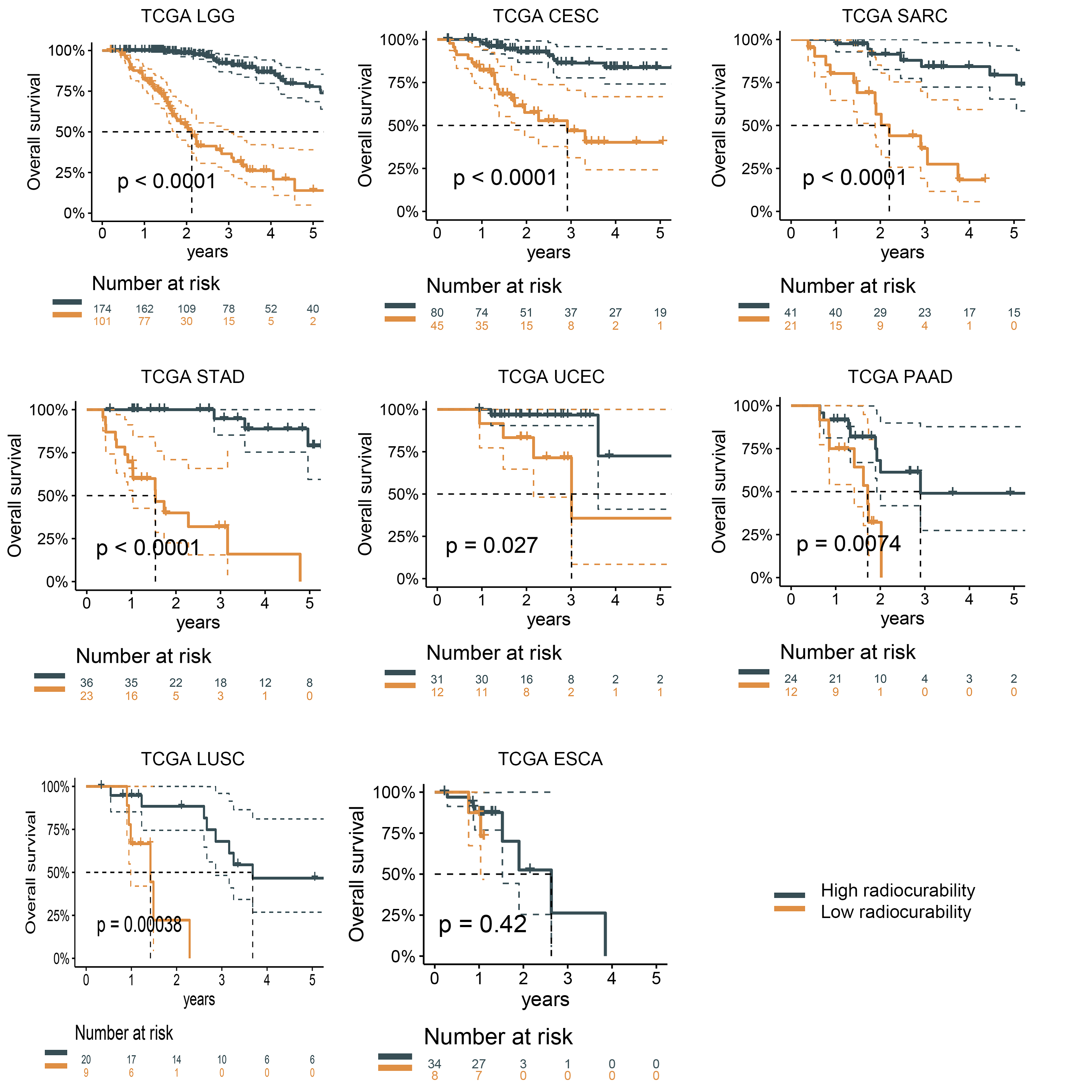
**

**Supplementary Fig. 11. Survival curves of high and low radiocurability groups in TCGA cohorts.**

**
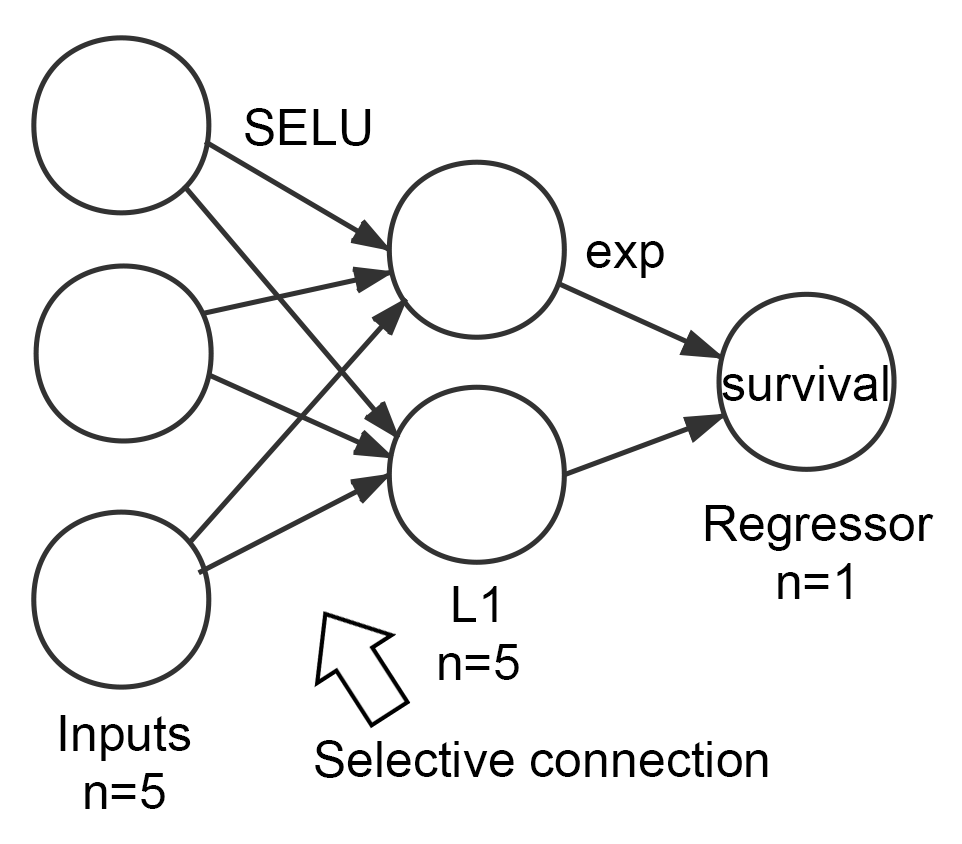
**

**Supplementary Fig. 12. ANN-SCGP with 1 hidden layer.**


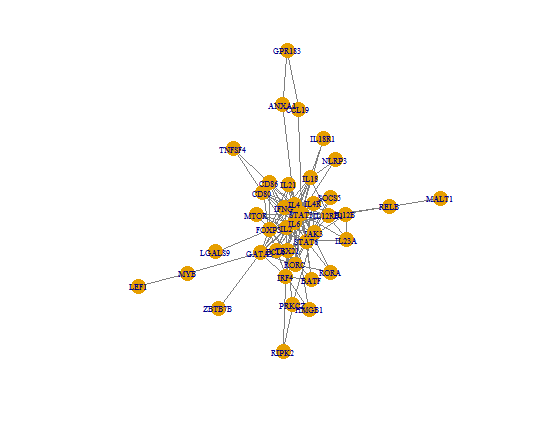


**Supplementary Fig. 13. Priori gene interactions via STRING.**


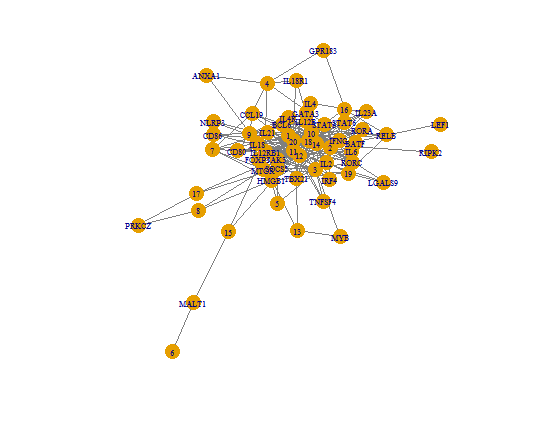


**Supplementary Fig. 14. Gene-deep node interactions in SCM.** SCM, selectively connected matrix.

**4. Additional Figures**

**Additional Figure S1. SF2 conformed to beta distribution.**


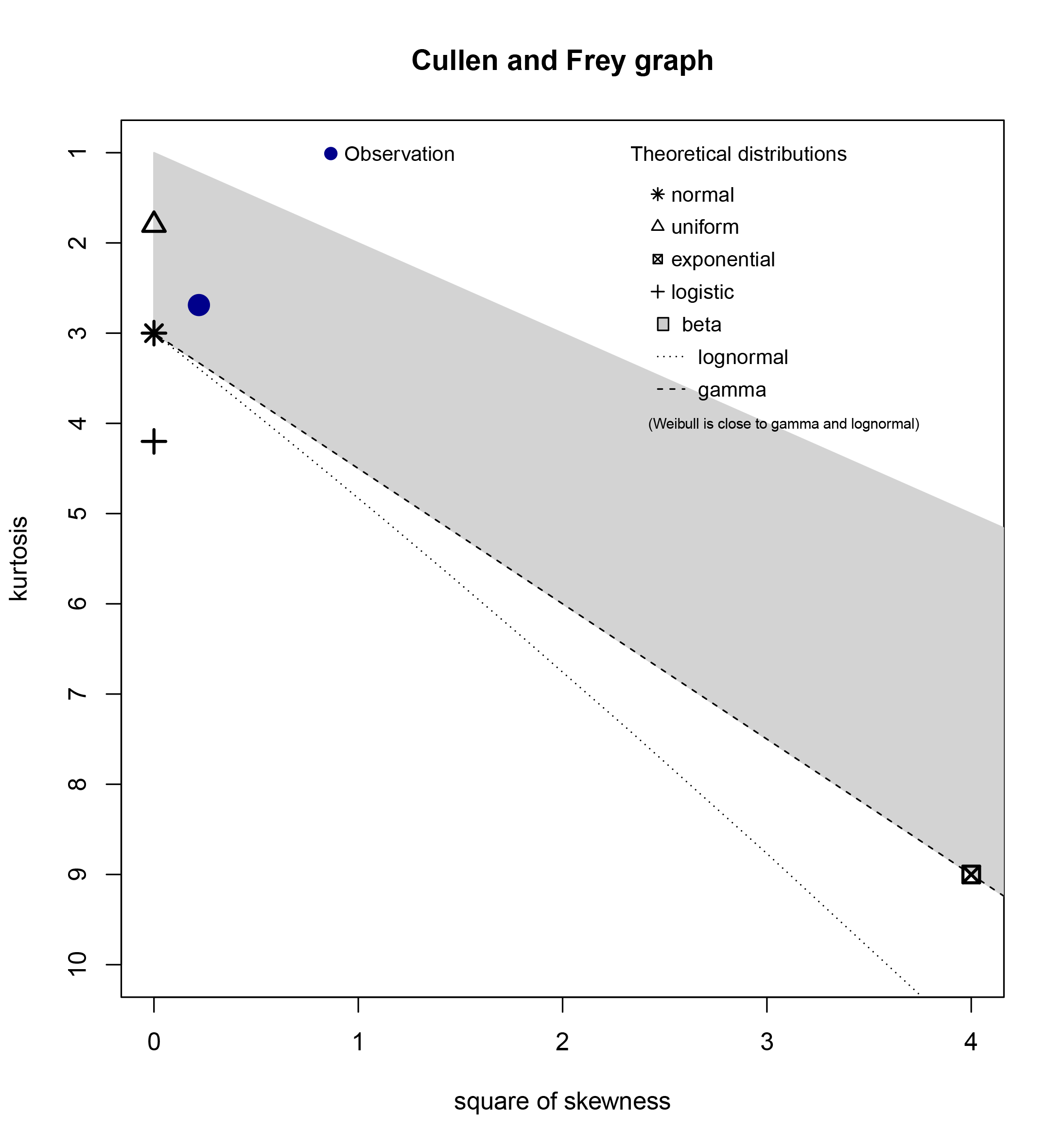


**5. Supplementary Tables**

**Supplementary Table 1. A summary table of previous gene model studies on radiosensitivity.**

| **Date** | **Title** | **Signatures** | **DOI** | **Prediction model** |
| --- | --- | --- | --- | --- |
| Aug-15 | Development and Validation of a Novel Radiosensitivity Signature in Human Breast Cancer | 147-gene signatures | 10.1158/1078-0432.CCR-14-2898 | Random Forest |
| Feb-17 | A genome-based model for adjusting radiotherapy dose (GARD): a retrospective, cohort-based study | 10-gene radiation-sensitivity index | 10.1016/S1470-2045(16)30648-9 | Rank-based regression |
| Aug-17 | Tumor Radiosensitivity is Associated with Immune Activation in Solid Tumors | 12-chemokine genes | 10.1016/j.ejca.2017.08.001 | PC1 |
| Jul-18 | Identification and validation of single-sample breast cancer radiosensitivity gene expression predictors | 284-gene signatures | 10.1186/s13058-018-0978-y | k-top scoring pairs algorithm |
| Apr-20 | A multiple genomic data fused SF2 prediction model, signature identification, and gene regulatory network inference for personalized radiotherapy | 108-mRNA expression values; 5-CNV signatures | 10.1177/1533033820909112 | Partial least squares; SVM |
| Aug-20 | Prediction of Radiosensitivity in Head and Neck Squamous Cell Carcinoma Based on Multiple Omics Data | 12-gene signatures | 10.3389/fgene.2020.00960 | Cox regression |
| Nov-20 | A novel methylation signature predicts radiotherapy sensitivity in glioma | 5-methylation probe | 10.1038/s41598-020-77259-9 | LASSO Cox regression |
| May-21 | Integration of machine learning and genome-scale metabolic modeling identifies multi-omics biomarkers for radiation resistance | Multi-omics | 10.1038/s41467-021-22989-1 | Ensemble-based machine learning classifiers |
| Jul-21 | Development and Validation of a Radiosensitivity Prediction Model for Lower Grade Glioma Based on Spike-and-Slab Lasso | 3-gene signatures | 10.3389/fonc.2021.701500 | spike-and-slab Lasso |
| Aug-21 | Development and validation of genomic predictors of radiation sensitivity using preclinical data | 30-gene signatures | 10.1186/s12885-021-08652-4 | Linear regression; ElasticNet |
| Sep-21 | Identification and Prognostic Value Exploration of Radiotherapy Sensitivity-Associated Genes in Non-Small-Cell Lung Cancer | 8-gene signatures | 10.1155/2021/5963868 | LASSO Cox regression |
| Feb-22 | A Radiosensitivity Prediction Model Developed Based on Weighted Correlation Network Analysis of Hypoxia Genes for Lower-Grade Glioma | 12-hypoxia genes | 10.3389/fonc.2022.757686 | LASSO Cox regression |
| Apr-22 | Development and validation of an immune-related gene signature for predicting the radiosensitivity of lower-grade gliomas | 21-immune-related gene signatures | 10.1038/s41598-022-10601-5 | LASSO Cox regression |
| May-22 | Construction of a novel radiosensitivity- and ferroptosis-associated gene signature for prognosis prediction in gliomas | 7-gene signatures | 10.7150/jca.72893 | LASSO Cox regression |
| May-22 | Prediction of Response to Radiotherapy by Characterizing the Transcriptomic Features in Clinical Tumor Samples across 15 Cancer Types | 100-gene signatures | 10.1155/2022/5443709 | Binary Logistic Regression |

**Supplementary Table 2. Radiation-related signatures.**

| **Gene** | **Type** | **Gene** | **Type** | **Gene** | **Type** | **Gene** | **Type** |
| --- | --- | --- | --- | --- | --- | --- | --- |
| ADM | Exp | BMI1 | Meth | PLEKHN1 | CNA | NOTCH1 | Mut |
| ARNTL | Exp | CDC20 | Meth | ISG15 | CNA | TTC28 | Mut |
| BIN3 | Exp | CDCA2 | Meth | MIR200B | CNA | ATRX | Mut |
| CABLES1 | Exp | CEP70 | Meth | AURKAIP1 | CNA | CENPE | Mut |
| CCNJL | Exp | CHMP4C | Meth | CCNL2 | CNA | CEP57 | Mut |
| CEACAM1 | Exp | ENSA | Meth | CDK11B | CNA | ERBB2 | Mut |
| CPEB2 | Exp | GADD45GIP1 | Meth | CDK11A | CNA | HSPG2 | Mut |
| DTX3L | Exp | GDPD5 | Meth | GNB1 | CNA | IQGAP1 | Mut |
| FANCF | Exp | INO80 | Meth | WRAP73 | CNA | KMT2E | Mut |
| FAP | Exp | NDE1 | Meth | TP73 | CNA | NFE2L2 | Mut |
| FOSL1 | Exp | PHGDH | Meth | MEOX2 | CNA | PLAT | Mut |
| GLI3 | Exp | PUM1 | Meth | AHR | CNA | PRKD2 | Mut |
| IQGAP1 | Exp | UBE2L3 | Meth | HDAC9 | CNA | ROCK2 | Mut |
| LATS2 | Exp | ANXA11 | Meth | TWIST1 | CNA | RPS6KA2 | Mut |
| LEF1 | Exp | ARHGEF10 | Meth | KLHL9 | CNA | SETX | Mut |
| LOXL2 | Exp | ATF5 | Meth | CXCL12 | CNA | SMAD4 | Mut |
| MAD1L1 | Exp | AXIN2 | Meth | RASSF4 | CNA | SND1 | Mut |
| MMP14 | Exp | BRSK1 | Meth | UBE2D1 | CNA | UBR5 | Mut |
| MNS1 | Exp | CDC14B | Meth | ANK3 | CNA |  |  |
| MX2 | Exp | CDK18 | Meth | SIRT1 | CNA |  |  |
| NEK10 | Exp | CDK5R1 | Meth | DNA2 | CNA |  |  |
| NPAS2 | Exp | CDK6 | Meth | NODAL | CNA |  |  |
| PARP9 | Exp | DCTN2 | Meth | C10orf99 | CNA |  |  |
| PLAT | Exp | DNMT3A | Meth | MMRN2 | CNA |  |  |
| PLOD2 | Exp | GEM | Meth | KLLN | CNA |  |  |
| PRKCE | Exp | GPR3 | Meth | PTEN | CNA |  |  |
| RASSF4 | Exp | LOXL2 | Meth | KIF20B | CNA |  |  |
| RUNX1 | Exp | MAPK13 | Meth | ANKRD1 | CNA |  |  |
| S100B | Exp | MDK | Meth | KIF11 | CNA |  |  |
| SERPINE1 | Exp | NES | Meth | HHEX | CNA |  |  |
| SETDB2 | Exp | PDK1 | Meth | CEP55 | CNA |  |  |
| SIPA1 | Exp | PRKCG | Meth | HELLS | CNA |  |  |
| SNAI2 | Exp | RARA | Meth | PDLIM1 | CNA |  |  |
| SP100 | Exp | RPTOR | Meth | CCNJ | CNA |  |  |
| SPRED1 | Exp | RRAS | Meth | DNTT | CNA |  |  |
| SPRY2 | Exp | SIX2 | Meth | MMS19 | CNA |  |  |
| TUBB2A | Exp | SOX18 | Meth | AVPI1 | CNA |  |  |
| WHAMM | Exp | TBX3 | Meth | MARVELD1 | CNA |  |  |
| ZNF268 | Exp | TH | Meth | HIF1AN | CNA |  |  |
| AGTRAP | Exp | TP73 | Meth | LZTS2 | CNA |  |  |
| ANGPT2 | Exp | TRRAP | Meth | BTRC | CNA |  |  |
| ANXA1 | Exp | TSPAN12 | Meth | POLL | CNA |  |  |
| ATP1B1 | Exp | TUBA1C | Meth | FGF8 | CNA |  |  |
| BANF1 | Exp | TUBA4A | Meth | NOLC1 | CNA |  |  |
| BCAT1 | Exp | VASN | Meth | GBF1 | CNA |  |  |
| BOD1 | Exp | WNT2B | Meth | FBXL15 | CNA |  |  |
| CALR | Exp | YAP1 | Meth | ACTR1A | CNA |  |  |
| CASP2 | Exp | ZFHX3 | Meth | ARL3 | CNA |  |  |
| CAV2 | Exp |  |  | PCGF6 | CNA |  |  |
| CCNA2 | Exp |  |  | SFR1 | CNA |  |  |
| CCND1 | Exp |  |  | PDCD4 | CNA |  |  |
| CCNI | Exp |  |  | TCF7L2 | CNA |  |  |
| CCNT2 | Exp |  |  | DCLRE1A | CNA |  |  |
| CCNYL1 | Exp |  |  | TDRD1 | CNA |  |  |
| CDK6 | Exp |  |  | AFAP1L2 | CNA |  |  |
| CDKN1B | Exp |  |  | VAX1 | CNA |  |  |
| CHEK1 | Exp |  |  | GRK5 | CNA |  |  |
| CLIC1 | Exp |  |  | MCMBP | CNA |  |  |
| DAGLA | Exp |  |  | FGFR2 | CNA |  |  |
| DDIT4 | Exp |  |  | NSMCE4A | CNA |  |  |
| DNMT3A | Exp |  |  | TACC2 | CNA |  |  |
| DSCC1 | Exp |  |  | CUZD1 | CNA |  |  |
| E2F6 | Exp |  |  | BUB3 | CNA |  |  |
| ECT2 | Exp |  |  | BCCIP | CNA |  |  |
| EMILIN1 | Exp |  |  | ADAM12 | CNA |  |  |
| ENDOG | Exp |  |  | MKI67 | CNA |  |  |
| ERAP1 | Exp |  |  | SUPT16H | CNA |  |  |
| ERN1 | Exp |  |  | METTL3 | CNA |  |  |
| EVI5 | Exp |  |  | MMP14 | CNA |  |  |
| EXO1 | Exp |  |  | AJUBA | CNA |  |  |
| FAM83D | Exp |  |  | MIR208A | CNA |  |  |
| FGFR1 | Exp |  |  | PSME1 | CNA |  |  |
| GATA3 | Exp |  |  | PSME2 | CNA |  |  |
| GINS4 | Exp |  |  | REC8 | CNA |  |  |
| GIPC1 | Exp |  |  | CHMP4A | CNA |  |  |
| HP1BP3 | Exp |  |  | TGM1 | CNA |  |  |
| INSM2 | Exp |  |  | CMA1 | CNA |  |  |
| INTS3 | Exp |  |  | EGLN3 | CNA |  |  |
| ISG15 | Exp |  |  | PSMA6 | CNA |  |  |
| ITGB8 | Exp |  |  | INSM2 | CNA |  |  |
| KIAA0753 | Exp |  |  | FOXA1 | CNA |  |  |
| KRIT1 | Exp |  |  | PSMC6 | CNA |  |  |
| LIG3 | Exp |  |  | BMP4 | CNA |  |  |
| MAGI2 | Exp |  |  | CDKN3 | CNA |  |  |
| MCM3 | Exp |  |  | CGRRF1 | CNA |  |  |
| MND1 | Exp |  |  | WDHD1 | CNA |  |  |
| MYC | Exp |  |  | DLGAP5 | CNA |  |  |
| NDE1 | Exp |  |  | PSMA3 | CNA |  |  |
| NUMB | Exp |  |  | DACT1 | CNA |  |  |
| PKMYT1 | Exp |  |  | PPM1A | CNA |  |  |
| PLOD1 | Exp |  |  | C14orf39 | CNA |  |  |
| PLXNB2 | Exp |  |  | MNAT1 | CNA |  |  |
| POGZ | Exp |  |  | SRSF5 | CNA |  |  |
| PSMB2 | Exp |  |  | SLC8A3 | CNA |  |  |
| RAD54B | Exp |  |  | SYNJ2BP | CNA |  |  |
| RAD9B | Exp |  |  | SETMAR | CNA |  |  |
| REEP4 | Exp |  |  | ITPR1 | CNA |  |  |
| RPAIN | Exp |  |  | ARL8B | CNA |  |  |
| RRM2 | Exp |  |  | CAMK1 | CNA |  |  |
| SERPINF1 | Exp |  |  | OGG1 | CNA |  |  |
| SFN | Exp |  |  | FANCD2 | CNA |  |  |
| SH3GLB1 | Exp |  |  | VHL | CNA |  |  |
| SMC3 | Exp |  |  | FBXL18 | CNA |  |  |
| SP1 | Exp |  |  | PMS2 | CNA |  |  |
| STARD13 | Exp |  |  | DAGLB | CNA |  |  |
| STOX1 | Exp |  |  | CBX3 | CNA |  |  |
| SUSD2 | Exp |  |  |  |  |  |  |
| TEAD3 | Exp |  |  |  |  |  |  |
| TOM1L1 | Exp |  |  |  |  |  |  |
| TRIM71 | Exp |  |  |  |  |  |  |
| TUBA1C | Exp |  |  |  |  |  |  |
| TUBA3E | Exp |  |  |  |  |  |  |
| TWIST1 | Exp |  |  |  |  |  |  |
| UBE2E2 | Exp |  |  |  |  |  |  |
| WIZ | Exp |  |  |  |  |  |  |
| ZMYND11 | Exp |  |  |  |  |  |  |

**Supplementary Table 3. Comparison of SF2 prediction in the dataset from the He's study.**

| **Methods** | **Training (RMSE)** | | | | **Testing (RMSE)** | | | |
| --- | --- | --- | --- | --- | --- | --- | --- | --- |
|  | **Mean** | **CV1** | **CV2** | **CV3** | **Mean** | **CV1** | **CV2** | **CV3** |
| ANN-SCGP | 0.0717 | 0.0634 | 0.0821 | 0.0696 | **0.1282** | **0.1414** | **0.1152** | **0.1281** |
| SVM | 0.0670 | 0.0599 | 0.0809 | 0.0601 | 0.1764 | 0.1805 | 0.1913 | 0.1573 |
| PLS | **0.0004** | **0.0009** | **0.0002** | **0.0002** | 0.1587 | 0.1580 | 0.1286 | 0.1894 |

**Supplementary Table 4. Multivariate Cox regression analysis in TCGA patients with RT in cross validation 1.**

| **Cohorts** | **Event/N** | **CV** | **Beta (P value)** | | | |
| --- | --- | --- | --- | --- | --- | --- |
|  |  |  | **ANN-SCGP** | **Age** | **Gender** | **Stage** |
| LGG | 90/275 | Training | -2.4359(1.21e-11) | 0.02135(0.245) | 0.37887(0.284) | NA |
|  |  | Testing | -2.72493(1.58e-10) | -0.00333(0.840) | 0.16833(0.644) | NA |
| HNSC | 107/264 | Training | -2.559537(4.53e-12) | 0.007341(0.6430) | -0.463480(0.2690) | 0.680431(0.0182) |
|  |  | Testing | -0.068410(0.09) | 0.016922(0.324) | 0.008058(0.983) | 0.217717(0.428) |
| CESC | 29/125 | Training | -4.52727(6.56e-05) | 0.02422(0.168) | NA | NA |
|  |  | Testing | -0.21271(0.137) | 0.00910(0.631) | NA | NA |
| SARC | 22/62 | Training | -5.674635(0.00793) | 0.069319(0.08813) | 2.390955(0.07355) | NA |
|  |  | Testing | -0.29270(0.0206) | 0.03347(0.1580) | -1.07301(0.1226) | NA |
| STAD | 18/59 | Training | -3.2701(0.0109) | -0.1425(0.1682) | 0.1295(0.9191) | -2.1217(0.0508) |
|  |  | Testing | -2.38398(0.00537) | -0.03798(0.35487) | 1.87833(0.11282) | 0.76494(0.39036) |
| UCEC | 7/43 | Training | -6.846(0.999) | -1.041(1.000) | NA | NA |
|  |  | Testing | -0.41647(0.101) | -0.05172(0.424) | NA | NA |
| ESCA | 10/42 | Training | -1.405(0.996) | -9.745e-01(0.998) | 2.153e+02(0.999) | 8.055(0.999) |
|  |  | Testing | -2.070(0.289) | 2.189e-02(0.805) | -1.817e+01(0.999) | 8.532e-01(0.308) |
| LUAD | 23/38 | Training | -1.738e+02(0.985) | 2.573e(0.991) | 2.228(1.000) | 2.587e+01(0.999) |
|  |  | Testing | -0.24857(0.498) | -0.02897(0.499) | -0.51096(0.545) | 0.60639(0.280) |
| PAAD | 16/36 | Training | -1.174e+01(0.0326) | -2.674e-02(0.6875) | 1.572(0.3371) | 1.006e+01(0.9997) |
|  |  | Testing | -1.39491(0.121) | 0.05869(0.348) | 0.09353(0.913) | -0.22930(0.855) |
| LUSC | 16/29 | Training | -1.992e+02(0.967) | -6.688e-01(0.993) | 6.922e+01(0.969) | -2.238e+01(0.985) |
|  |  | Testing | -0.78720(0.208) | 0.02989(0.622) | 0.30840(0.829) | 0.65076(0.322) |

**Supplementary Table 5. Multivariate Cox regression analysis in TCGA patients with RT in cross validation 2.**

| **Cohorts** | **Event/N** | **CV** | **Beta (P value)** | | | |
| --- | --- | --- | --- | --- | --- | --- |
|  |  |  | **ANN-SCGP** | **Age** | **Gender** | **Stage** |
| LGG | 90/275 | Training | -0.21197(0.002692) | 0.04594(0.000246) | 0.10240(0.744504) | NA |
|  |  | Testing | -0.28869(4.33e-06) | 0.04999(0.0002) | -0.01971(0.9492) | NA |
| HNSC | 107/264 | Training | -2.74784(1.81e-11) | 0.03003(0.1673) | -0.85303(0.0555) | 0.23023(0.4354) |
|  |  | Testing | -0.1110487(0.116) | 0.0001068(0.994) | -0.1236459(0.755) | 0.3436872(0.141) |
| CESC | 29/125 | Training | -3.01052(0.000179) | 0.02102(0.241269) | NA | NA |
|  |  | Testing | -0.12244(0.174) | 0.01439(0.446) | NA | NA |
| SARC | 22/62 | Training | -9.927(0.0674) | 7.081e-02(0.2556) | -2.312(0.4952) | NA |
|  |  | Testing | -0.04000(0.762) | 0.03629(0.208) | 0.37290(0.586) | NA |
| STAD | 18/59 | Training | -1.226e+01(0.115) | -1.529e-01(0.327) | 1.869(0.494) | -9.796e-01(0.542) |
|  |  | Testing | -0.51289(0.0561) | -0.01023(0.8373) | -0.25347(0.7811) | -0.21398(0.6917) |
| UCEC | 7/43 | Training | -2.92607(0.0659) | 0.03542(0.7190) | NA | NA |
|  |  | Testing | -0.7767(0.257) | -0.1134(0.399) | NA | NA |
| ESCA | 10/42 | Training | -1.821(0.336) | 1.785e-02(0.821) | -1.941e+01(0.999) | 7.241e-01(0.372) |
|  |  | Testing | 1.756(0.401) | 3.820e-02(0.822) | -1.728e+01(1.000) | -3.823e-01(0.922) |
| LUAD | 23/38 | Training | -1.71145(0.0234) | 0.01767(0.6813) | -0.07326(0.9259) | 0.63159(0.2683) |
|  |  | Testing | -0.48548(0.318) | 0.05607(0.210) | 1.19725(0.208) | 0.51473(0.281) |
| PAAD | 16/36 | Training | -8.440(0.0170) | 2.084e-01(0.0446) | -5.748e-01(0.6027) | -1.574e+01(0.9963) |
|  |  | Testing | -5.644e-01(0.360) | 9.078e-02(0.105) | 4.692e-01(0.598) | 1.623e+01(0.999) |
| LUSC | 16/29 | Training | -3.46129(0.139) | -0.07381(0.624) | 1.65947(0.677) | 0.62387(0.498) |
|  |  | Testing | -0.91519(0.0768) | 0.04037(0.5051) | -1.65472(0.1658) | -0.49974(0.5179) |

**Supplementary Table 6. AUC of T value of C-index between high-low occlusion score groups in each cut-off point.**

| **TCGA datasets** | **AUC** | **Sig. negative nodes** | **Sig. positive nodes** | **binom.test** |
| --- | --- | --- | --- | --- |
| LGG | -24.36266 | 12 | 2 | 0.01294 |
| HNSC | -538.019 | 117 | 0 | <0.0001 |
| CESC | -800.383 | 232 | 0 | <0.0001 |
| SARC | -28.99692 | 0 | 33 | <0.0001 |
| STAD | -541.1127 | 145 | 0 | <0.0001 |
| UCEC | -600.5034 | 82 | 0 | <0.0001 |
| ESCA | -516.8688 | 134 | 0 | <0.0001 |
| LUAD | -705.3521 | 100 | 0 | <0.0001 |
| PAAD | -212.5012 | 22 | 1 | <0.0001 |
| LUSC | -450.4359 | 111 | 0 | <0.0001 |

**Supplementary Table 7. AUC of T value of HR of HRD & mutation scores in each cut-off point.**

| **Type** | **TCGA datasets** | **AUC** | **Sig. negative nodes** | **Sig. positive nodes** | **binom.test** |
| --- | --- | --- | --- | --- | --- |
| HRD | LGG | 11.08566 | 0 | 1 | 1 |
| HRD | HNSC | 28.92 | 0 | 9 | 0.003906 |
| HRD | CESC | 10.58685 | 0 | 2 | 0.5 |
| HRD | SARC | 6.824042 | 0 | 0 | 1 |
| HRD | STAD | 18.64927 | 0 | 0 | 1 |
| HRD | UCEC | 19.07809 | 0 | 0 | 1 |
| HRD | ESCA | 3.005031 | 0 | 0 | 1 |
| HRD | LUAD | -2.749514 | 0 | 0 | 1 |
| HRD | PAAD | 1.313969 | 0 | 0 | 1 |
| HRD | LUSC | -0.2406331 | 0 | 0 | 1 |
| Mut counts | LGG | 51.61972 | 0 | 34 | <0.0001 |
| Mut counts | HNSC | 13.95585 | 0 | 0 | 1 |
| Mut counts | CESC | -29.32817 | 11 | 0 | 0.0009766 |
| Mut counts | SARC | 7.202621 | 5 | 0 | 0.0625 |
| Mut counts | STAD | -19.66597 | 0 | 0 | 1 |
| Mut counts | UCEC | -28.57444 | 12 | 0 | 0.0004883 |
| Mut counts | ESCA | -0.9104336 | 0 | 0 | 1 |
| Mut counts | LUAD | 3.351997 | 0 | 0 | 1 |
| Mut counts | PAAD | -1.895233 | 1 | 0 | 1 |
| Mut counts | LUSC | 7.455927 | 0 | 0 | 1 |

**Supplementary Table 8. AUC of T value of HR of immune infiltration scores in each cut-off point.**

| **Immune infiltration** | **TCGA datasets** | **AUC** | **Sig. negative nodes** | **Sig. positive nodes** | **binom.test** |
| --- | --- | --- | --- | --- | --- |
| T cell | LGG | 355.081 | 254 | 0 | <0.0001 |
| T cell | HNSC | -103.0138 | 0 | 149 | <0.0001 |
| T cell | CESC | -61.52446 | 0 | 57 | <0.0001 |
| T cell | SARC | -25.80408 | 0 | 3 | 0.25 |
| T cell | STAD | -20.77081 | 0 | 19 | <0.0001 |
| T cell | UCEC | 33.0902 | 0 | 0 | 1 |
| T cell | ESCA | 84.32578 | 7 | 0 | 0.01563 |
| T cell | LUAD | -11.3137 | 0 | 5 | 0.0625 |
| T cell | PAAD | -0.4524114 | 0 | 0 | 1 |
| T cell | LUSC | 13.3929 | 1 | 0 | 1 |
| B lineage | LGG | 256.357 | 116 | 0 | <0.0001 |
| B lineage | HNSC | -116.2313 | 0 | 185 | <0.0001 |
| B lineage | CESC | -43.51881 | 0 | 0 | 1 |
| B lineage | SARC | -22.45944 | 0 | 1 | 1 |
| B lineage | STAD | -15.38969 | 0 | 0 | 1 |
| B lineage | UCEC | 4.295465 | 0 | 0 | 1 |
| B lineage | ESCA | 24.51874 | 0 | 0 | 1 |
| B lineage | LUAD | -8.445924 | 0 | 0 | 1 |
| B lineage | PAAD | -11.65995 | 0 | 0 | 1 |
| B lineage | LUSC | 0.9650578 | 0 | 0 | 1 |
| NK cell | LGG | -7.591914 | 0 | 0 | 1 |
| NK cell | HNSC | -76.51571 | 0 | 107 | <0.0001 |
| NK cell | CESC | -58.53572 | 0 | 14 | 0.0001221 |
| NK cell | SARC | -24.09906 | 0 | 5 | 0.0625 |
| NK cell | STAD | -13.54189 | 0 | 2 | 0.5 |
| NK cell | UCEC | 40.38834 | 1 | 0 | 1 |
| NK cell | ESCA | 47.90175 | 1 | 0 | 1 |
| NK cell | LUAD | -8.146946 | 0 | 0 | 1 |
| NK cell | PAAD | 10.53585 | 0 | 0 | 1 |
| NK cell | LUSC | 0.5555503 | 0 | 0 | 1 |
| Myeloid dendritic cell | LGG | 22.95013 | 1 | 0 | 1 |
| Myeloid dendritic cell | HNSC | -81.2983 | 0 | 98 | <0.0001 |
| Myeloid dendritic cell | CESC | -61.14867 | 0 | 31 | <0.0001 |
| Myeloid dendritic cell | SARC | -27.19718 | 0 | 18 | <0.0001 |
| Myeloid dendritic cell | STAD | 17.78626 | 2 | 0 | 0.5 |
| Myeloid dendritic cell | UCEC | 30.33722 | 0 | 0 | 1 |
| Myeloid dendritic cell | ESCA | 19.95934 | 0 | 0 | 1 |
| Myeloid dendritic cell | LUAD | -9.876051 | 0 | 4 | 0.125 |
| Myeloid dendritic cell | PAAD | 19.8548 | 0 | 0 | 1 |
| Myeloid dendritic cell | LUSC | 0.8394804 | 0 | 0 | 1 |
| Monocytic lineage | LGG | 99.41738 | 17 | 3 | 0.002577 |
| Monocytic lineage | HNSC | 4.306438 | 19 | 0 | <0.0001 |
| Monocytic lineage | CESC | -42.91911 | 0 | 10 | 0.001953 |
| Monocytic lineage | SARC | -33.03111 | 0 | 20 | <0.0001 |
| Monocytic lineage | STAD | 19.308 | 1 | 0 | 1 |
| Monocytic lineage | UCEC | 57.03492 | 3 | 0 | 0.25 |
| Monocytic lineage | ESCA | 36.48314 | 3 | 0 | 0.25 |
| Monocytic lineage | LUAD | -6.357215 | 0 | 0 | 1 |
| Monocytic lineage | PAAD | 23.462 | 0 | 0 | 1 |
| Monocytic lineage | LUSC | 6.735082 | 0 | 0 | 1 |

**6. Additional Tables**

**Additional Table S1. The 82 CCLE cell lines with complete omics data and SF2 annotations from previous laboratory studies.**

| **Cell line** | **Origin_organs** | **Histology** | **TCGA_code** | **SF2** | **Discretized_value_O(Origin_organs)** | **Discretized_value_H(Histology)** |
| --- | --- | --- | --- | --- | --- | --- |
| 22RV1 | prostate | carcinoma | PRAD | 0.4 | 0 | 0 |
| 786O | kidney | carcinoma | KIRC | 0.457 | 0 | 0 |
| A375 | skin | malignant_melanoma | SKCM | 0.4585 | 0 | 0 |
| A498 | kidney | carcinoma | KIRC | 0.57 | 0 | 0 |
| A549 | lung | carcinoma | LUAD | 0.6686 | 0 | 0 |
| ACHN | kidney | carcinoma | KIRC | 0.684 | 0 | 0 |
| CACO2 | large_intestine | carcinoma | COAD/READ | 0.72 | 0 | 0 |
| CAKI1 | kidney | carcinoma | KIRC | 0.368 | 0 | 0 |
| COLO320 | large_intestine | carcinoma | COAD/READ | 0.34 | 0 | 0 |
| D283MED | central_nervous_system | neurogenic_tumor | MB | 0.245 | 0 | 0 |
| DU145 | prostate | carcinoma | PRAD | 0.6324 | 0 | 0 |
| EKVX | lung | carcinoma | LUAD | 0.82 | 0 | 0 |
| FADU | upper_aerodigestive_tract | carcinoma | HNSC | 0.619 | 0 | 0 |
| HCC2429 | lung | carcinoma | NA | 0.51 | 0 | 0 |
| HCT116 | large_intestine | carcinoma | COAD/READ | 0.34345 | 0 | 0 |
| HCT15 | large_intestine | carcinoma | COAD/READ | 0.449666667 | 0 | 0 |
| HEC1A | endometrium | carcinoma | UCEC | 0.88 | 3 | 0 |
| HEC1B | endometrium | carcinoma | UCEC | 0.94 | 3 | 0 |
| HELA | Cervix | carcinoma | CESC | 0.47075 | 0 | 0 |
| HEPG2 | liver | carcinoma | LIHC | 0.624433333 | 0 | 0 |
| HL60 | haematopoietic_and_lymphoid_tissue | Lymphoid_hematopoietic_neoplasm | LAML | 0.2 | -2 | -1 |
| HOP62 | lung | carcinoma | LUAD | 0.224 | 0 | 0 |
| HOP92 | lung | carcinoma | NA | 0.36 | 0 | 0 |
| HS578T | breast | carcinoma | BRCA | 0.643333333 | 0 | 0 |
| HT1080 | soft_tissue | sarcoma | SARC | 0.78 | 0 | 1 |
| HT1376 | urinary_tract | carcinoma | BLCA | 0.835 | 1 | 0 |
| HT29 | large_intestine | carcinoma | COAD/READ | 0.680142857 | 0 | 0 |
| IGROV1 | ovary | carcinoma | OV | 0.3895 | 0 | 0 |
| IMR32 | autonomic_ganglia | neurogenic_tumor | NBL | 0.25 | -1 | 0 |
| J82 | urinary_tract | carcinoma | BLCA | 0.615 | 1 | 0 |
| K562 | haematopoietic_and_lymphoid_tissue | Lymphoid_hematopoietic_neoplasm | LCML | 0.05 | -2 | -1 |
| KELLY | autonomic_ganglia | neurogenic_tumor | NBL | 0.2 | -1 | 0 |
| KYSE150 | oesophagus | carcinoma | ESCA | 0.69 | 2 | 0 |
| KYSE30 | oesophagus | carcinoma | ESCA | 0.77 | 2 | 0 |
| KYSE450 | oesophagus | carcinoma | ESCA | 0.81 | 2 | 0 |
| LOVO | large_intestine | carcinoma | COAD/READ | 0.456666667 | 0 | 0 |
| LOXIMVI | skin | malignant_melanoma | SKCM | 0.68 | 0 | 0 |
| LS1034 | large_intestine | carcinoma | COAD/READ | 0.81 | 0 | 0 |
| LS411N | large_intestine | carcinoma | COAD/READ | 0.32 | 0 | 0 |
| LS513 | large_intestine | carcinoma | COAD/READ | 0.49 | 0 | 0 |
| M059K | central_nervous_system | neurogenic_tumor | GBM | 0.64 | 0 | 0 |
| MALME3M | skin | malignant_melanoma | SKCM | 0.6835 | 0 | 0 |
| MCF7 | breast | carcinoma | BRCA | 0.64825 | 0 | 0 |
| MDAMB231 | breast | carcinoma | BRCA | 0.4948 | 0 | 0 |
| MDAMB453 | breast | carcinoma | BRCA | 0.37 | 0 | 0 |
| MDAMB468 | breast | carcinoma | BRCA | 0.532 | 0 | 0 |
| MEWO | skin | malignant_melanoma | SKCM | 0.3925 | 0 | 0 |
| MIAPACA2 | pancreas | carcinoma | PAAD | 0.66 | 0 | 0 |
| ML1 | thyroid | carcinoma | THCA | 0.038 | 0 | 0 |
| NCIH1299 | lung | carcinoma | NA | 0.69 | 0 | 0 |
| NCIH2009 | lung | carcinoma | LUAD | 0.62 | 0 | 0 |
| NCIH226 | lung | carcinoma | LUSC | 0.63 | 0 | 0 |
| NCIH23 | lung | carcinoma | LUAD | 0.119333333 | 0 | 0 |
| NCIH322 | lung | carcinoma | LUAD | 0.59 | 0 | 0 |
| NCIH358 | lung | carcinoma | LUAD | 0.433 | 0 | 0 |
| NCIH520 | lung | carcinoma | LUSC | 0.3985 | 0 | 0 |
| NCIH522 | lung | carcinoma | LUAD | 0.43 | 0 | 0 |
| NCIH596 | lung | carcinoma | LUAD | 0.4 | 0 | 0 |
| NCIH661 | lung | carcinoma | NA | 0.593 | 0 | 0 |
| NIHOVCAR3 | ovary | carcinoma | OV | 0.5635 | 0 | 0 |
| OAW42 | ovary | carcinoma | OV | 0.22 | 0 | 0 |
| OSRC2 | kidney | carcinoma | KIRC | 0.762 | 0 | 0 |
| OVCAR4 | ovary | carcinoma | OV | 0.2655 | 0 | 0 |
| OVCAR8 | ovary | carcinoma | OV | 0.5805 | 0 | 0 |
| PANC1 | pancreas | carcinoma | PAAD | 0.729 | 0 | 0 |
| PC3 | prostate | carcinoma | PRAD | 0.542222222 | 0 | 0 |
| RD | soft_tissue | sarcoma | SARC | 0.81 | 0 | 1 |
| RERFLCAI | lung | carcinoma | LUSC | 0.679 | 0 | 0 |
| RKO | large_intestine | carcinoma | COAD/READ | 0.515 | 0 | 0 |
| RPMI7951 | skin | malignant_melanoma | SKCM | 0.56 | 0 | 0 |
| RPMI8226 | haematopoietic_and_lymphoid_tissue | Lymphoid_hematopoietic_neoplasm | MM | 0.0995 | -2 | -1 |
| RT112 | urinary_tract | carcinoma | BLCA | 0.634666667 | 1 | 0 |
| SF268 | central_nervous_system | neurogenic_tumor | GBM | 0.475 | 0 | 0 |
| SF295 | central_nervous_system | neurogenic_tumor | GBM | 0.68 | 0 | 0 |
| SF539 | central_nervous_system | neurogenic_tumor | LGG | 0.685 | 0 | 0 |
| SF767 | central_nervous_system | neurogenic_tumor | LGG | 0.32 | 0 | 0 |
| SIHA | Cervix | carcinoma | CESC | 0.579333333 | 0 | 0 |
| SKMEL28 | skin | malignant_melanoma | SKCM | 0.845 | 0 | 0 |
| SKMEL3 | skin | malignant_melanoma | SKCM | 0.93 | 0 | 0 |
| SKMEL5 | skin | malignant_melanoma | SKCM | 0.786 | 0 | 0 |
| SKNBE2 | autonomic_ganglia | neurogenic_tumor | NBL | 0.53 | -1 | 0 |
| SKNSH | autonomic_ganglia | neurogenic_tumor | NBL | 0.19 | -1 | 0 |
| SKOV3 | ovary | carcinoma | OV | 0.78 | 0 | 0 |
| SNB75 | central_nervous_system | neurogenic_tumor | GBM | 0.63 | 0 | 0 |
| SW1990 | pancreas | carcinoma | PAAD | 0.517433333 | 0 | 0 |
| SW403 | large_intestine | carcinoma | COAD/READ | 0.5897 | 0 | 0 |
| SW480 | large_intestine | carcinoma | COAD/READ | 0.612925 | 0 | 0 |
| SW48 | large_intestine | carcinoma | COAD/READ | 0.1595 | 0 | 0 |
| T24 | urinary_tract | carcinoma | BLCA | 0.70725 | 1 | 0 |
| T47D | breast | carcinoma | BRCA | 0.466666667 | 0 | 0 |
| T98G | central_nervous_system | neurogenic_tumor | GBM | 0.673333333 | 0 | 0 |
| TCCSUP | urinary_tract | carcinoma | BLCA | 0.62 | 1 | 0 |
| TE1 | oesophagus | carcinoma | ESCA | 0.8 | 2 | 0 |
| U251MG | central_nervous_system | neurogenic_tumor | GBM | 0.582333333 | 0 | 0 |
| U2OS | bone | sarcoma | SARC | 0.883 | 0 | 1 |
| U87MG | central_nervous_system | neurogenic_tumor | GBM | 0.6 | 0 | 0 |
| UMUC3 | urinary_tract | carcinoma | BLCA | 0.825 | 1 | 0 |

**Additional Table S2. Simple ANN-SCGP model with PQC outperformed CPH in training and testing of all the 4 datasets after 3,000 iterations.**

| **Datasets** | **CPH (C-index)** | | **ANN-SCGP (C-index)** | | **PQC** | **NLPL** |
| --- | --- | --- | --- | --- | --- | --- |
|  | **Training** | **Testing** | **Training** | **Testing** | **Time (s)** | **Time (s)** |
| METABRIC (n=1904) | 0.6407634 | 0.6321975 | 0.641489229 | 0.637411292 | 1.03 | 10.88 |
| WHAS (n=1638) | 0.8211059 | 0.8165762 | 0.823976661 | 0.820384914 | 1.09 | 6.88 |
| SUPPORT (n=8873) | 0.5693584 | 0.58343 | 0.569966266 | 0.585386247 | 2.87 | 172.44 |
| GBSG (n=2232) | 0.6625285 | 0.6525791 | 0.665527617 | 0.668059396 | 1.14 | 9.75 |

**7. References**

1. Barretina J, Caponigro G, Stransky N, Venkatesan K, Margolin AA, Kim S, Wilson CJ, Lehár J, Kryukov GV, Sonkin D *et al*: **The Cancer Cell Line Encyclopedia enables predictive modelling of anticancer drug sensitivity**. *Nature* 2012, **483**(7391):603-607.

2. Garnett MJ, Edelman EJ, Heidorn SJ, Greenman CD, Dastur A, Lau KW, Greninger P, Thompson IR, Luo X, Soares J *et al*: **Systematic identification of genomic markers of drug sensitivity in cancer cells**. *Nature* 2012, **483**(7391):570-575.

3. Wagner GP, Kin K, Lynch VJ: **Measurement of mRNA abundance using RNA-seq data: RPKM measure is inconsistent among samples**. *Theory in biosciences = Theorie in den Biowissenschaften* 2012, **131**(4):281-285.

4. Newman AM, Liu CL, Green MR, Gentles AJ, Feng W, Xu Y, Hoang CD, Diehn M, Alizadeh AA: **Robust enumeration of cell subsets from tissue expression profiles**. *Nat Methods* 2015, **12**(5):453-457.

5. Marabita F, Almgren M, Lindholm ME, Ruhrmann S, Fagerström-Billai F, Jagodic M, Sundberg CJ, Ekström TJ, Teschendorff AE, Tegnér J *et al*: **An evaluation of analysis pipelines for DNA methylation profiling using the Illumina HumanMethylation450 BeadChip platform**. *Epigenetics* 2013, **8**(3):333-346.

6. Rashid A, Liu C, Sanli T, Tsiani E, Singh G, Bristow R, Dayes I, Lukka H, Wright J, Tsakiridis T: **Resveratrol enhances prostate cancer cell response to ionizing radiation. Modulation of the AMPK, Akt and mTOR pathways**. *Radiation oncology (London, England)* 2011, **6**:144.

7. Amundson SA, Do KT, Vinikoor LC, Lee RA, Koch-Paiz CA, Ahn J, Reimers M, Chen Y, Scudiero DA, Weinstein JN *et al*: **Integrating global gene expression and radiation survival parameters across the 60 cell lines of the National Cancer Institute Anticancer Drug Screen**. *Cancer research* 2008, **68**(2):415-424.

8. Munshi A, Tanaka T, Hobbs ML, Tucker SL, Richon VM, Meyn RE: **Vorinostat, a histone deacetylase inhibitor, enhances the response of human tumor cells to ionizing radiation through prolongation of gamma-H2AX foci**. *Molecular cancer therapeutics* 2006, **5**(8):1967-1974.

9. Munshi A, Kurland JF, Nishikawa T, Tanaka T, Hobbs ML, Tucker SL, Ismail S, Stevens C, Meyn RE: **Histone deacetylase inhibitors radiosensitize human melanoma cells by suppressing DNA repair activity**. *Clinical cancer research : an official journal of the American Association for Cancer Research* 2005, **11**(13):4912-4922.

10. Zhang C, Girard L, Das A, Chen S, Zheng G, Song K: **Nonlinear quantitative radiation sensitivity prediction model based on NCI-60 cancer cell lines**. *TheScientificWorldJournal* 2014, **2014**:903602-903602.

11. Ning S, Trisler K, Wessels BW, Knox SJ: **Radiobiologic studies of radioimmunotherapy and external beam radiotherapy in vitro and in vivo in human renal cell carcinoma xenografts**. *Cancer* 1997, **80**(12 Suppl):2519-2528.

12. Zhuang L, Yu S, Huang X, Cao Y, Xiong H: **Correlativity study between expression of DNA double-strand break repair protein and radiosensitivity of tumor cells**. *Frontiers of Medicine in China* 2009, **3**(1):26-29.

13. El-Ashmawy M, Delgado O, Cardentey A, Wright WE, Shay JW: **CDDO-Me protects normal lung and breast epithelial cells but not cancer cells from radiation**. *PloS one* 2014, **9**(12):e115600.

14. Sak A, Groneberg M, Stuschke M: **DNA-dependent protein kinase: effect on DSB repair, G2/M checkpoint and mode of cell death in NSCLC cell lines**. *International journal of radiation biology* 2019, **95**:1-37.

15. Zernickel E, Sak A, Riaz A, Klein D, Groneberg M, Stuschke M: **Targeting of BRM Sensitizes BRG1-Mutant Lung Cancer Cell Lines to Radiotherapy**. *Molecular cancer therapeutics* 2019, **18**(3):656-666.

16. Sun J, Liu NB, Zhuang HQ, Zhao LJ, Yuan ZY, Wang P: **Celecoxib-erlotinib combination treatment enhances radiosensitivity in A549 human lung cancer cell**. *Cancer Biomark* 2017, **19**(1):45-50.

17. Zhong X, Luo G, Zhou X, Luo W, Wu X, Zhong R, Wang Y, Xu F, Wang J: **Rad51 in regulating the radiosensitivity of non-small cell lung cancer with different epidermal growth factor receptor mutation status**. *Thoracic cancer* 2016, **7**(1):50-60.

18. Jiang L, Xiong XP, Hu CS, Ou ZL, Zhu GP, Ying HM: **In vitro and in vivo studies on radiobiological effects of prolonged fraction delivery time in A549 cells**. *J Radiat Res* 2013, **54**(2):230-234.

19. Zuo ZG, Yu ZQ, Gao XH, Wang H, Zhang C, Liu QZ, Han YF, Chen LP, Zhang XQ, Fu CG: **[Association of epithermal growth factor receptor expression and its downstream gene mutation status with radiosensitivity of colorectal carcinoma cell lines in vitro]**. *Zhonghua Wei Chang Wai Ke Za Zhi* 2013, **16**(8):753-758.

20. Warenius HM, Jones M, Jones MD, Browning PG, Seabra LA, Thompson CC: **Late G1 accumulation after 2 Gy of gamma-irradiation is related to endogenous Raf-1 protein expression and intrinsic radiosensitivity in human cells**. *Br J Cancer* 1998, **77**(8):1220-1228.

21. Bush C, McMillan TJ: **Micronucleus formation in human tumour cells: lack of correlation with radiosensitivity**. *Br J Cancer* 1993, **67**(1):102-106.

22. Whitaker SJ, Ung YC, McMillan TJ: **DNA double-strand break induction and rejoining as determinants of human tumour cell radiosensitivity. A pulsed-field gel electrophoresis study**. *International journal of radiation biology* 1995, **67**(1):7-18.

23. Wollin M, FitzGerald TJ, Santucci MA, Menon M, Longcope C, Reale F, Carlson J, Sakakeeny MA, Greenberger JS: **Radiosensitivity of human prostate cancer and malignant melanoma cell lines**. *Radiotherapy and oncology : journal of the European Society for Therapeutic Radiology and Oncology* 1989, **15**(3):285-293.

24. Brooks C, Sheu T, Bridges K, Mason K, Kuban D, Mathew P, Meyn R: **Preclinical evaluation of sunitinib, a multi-tyrosine kinase inhibitor, as a radiosensitizer for human prostate cancer**. *Radiat Oncol* 2012, **7**:154.

25. Elgqvist J, Timmermand OV, Larsson E, Strand SE: **Radiosensitivity of Prostate Cancer Cell Lines for Irradiation from Beta Particle-emitting Radionuclide ¹⁷⁷Lu Compared to Alpha Particles and Gamma Rays**. *Anticancer research* 2016, **36**(1):103-109.

26. Danielsson A, Karlsson E, Delle U, Helou K, Mercke C: **The biological effect of pentoxifylline on the survival of human head and neck cancer cells treated with continuous low and high dose-rate irradiation**. *J Cancer Res Clin Oncol* 2005, **131**(7):459-467.

27. DeLaney TF, Afridi N, Taghian AG, Sanders DA, Fuleihan NS, Faller DV, Nogueira CP: **13-cis-retinoic acid with alpha-2a-interferon enhances radiation cytotoxicity in head and neck squamous cell carcinoma in vitro**. *Cancer research* 1996, **56**(10):2277-2280.

28. Shao Y, Quan F, Li HH, Yao XB, Zhao Q, Zhao RM: **[The Radiosensitizing Effect of Resveratrol on Hopypharyngeal Carcinoma Cell Line FADU and its Effect on the Cell Cycle]**. *Zhongguo Zhong Xi Yi Jie He Za Zhi* 2015, **35**(6):699-703.

29. Huang MY, Wang JY, Chang HJ, Kuo CW, Tok TS, Lin SR: **CDC25A, VAV1, TP73, BRCA1 and ZAP70 gene overexpression correlates with radiation response in colorectal cancer**. *Oncol Rep* 2011, **25**(5):1297-1306.

30. Häggblad Sahlberg S, Spiegelberg D, Lennartsson J, Nygren P, Glimelius B, Stenerlöw B: **The effect of a dimeric Affibody molecule (ZEGFR:1907)2 targeting EGFR in combination with radiation in colon cancer cell lines**. *International journal of oncology* 2012, **40**(1):176-184.

31. Huang P, Taghian A, Hsu DW, Perez LA, Allam A, Duffy M, DaCosta A, Suit HD: **Spontaneous metastasis, proliferation characteristics and radiation sensitivity of fractionated irradiation recurrent and unirradiated human xenografts**. *Radiotherapy and oncology : journal of the European Society for Therapeutic Radiology and Oncology* 1996, **41**(1):73-81.

32. Nguyen HN, Sevin BU, Averette HE, Gottlieb CF, Perras J, Ramos R, Donato D, Penalver M: **The use of ATP bioluminescence assay and flow cytometry in predicting radiosensitivity of uterine cancer cell lines: correlation of radiotoxicity and cell cycle kinetics**. *Gynecol Oncol* 1992, **46**(1):88-96.

33. Deng YR, Jiang HP, Wu LF, Chen W, Lin D, Guo SQ: **[Role of specificity protein 1 in modulating radiosensitivity of cervical cancer cell lines]**. *Nan Fang Yi Ke Da Xue Xue Bao* 2016, **36**(9):1226-1230.

34. Tam KF, Ng TY, Liu SS, Tsang PC, Kwong PW, Ngan HY: **Potential application of the ATP cell viability assay in the measurement of intrinsic radiosensitivity in cervical cancer**. *Gynecol Oncol* 2005, **96**(3):765-770.

35. Ling ZH, Sun QQ, Zhang YW, Guan J, Ding Y, Chen LH: **[Effect of gemcitabine in enhancing the radiosensitivity of HepG2 hepatoma cells and the possible mechanism]**. *Nan Fang Yi Ke Da Xue Xue Bao* 2011, **31**(12):1993-1996.

36. Ma XJ, Chen XP: **[Relationship between the radiosensitivity of hepatic carcinoma cells and their survivin expression levels]**. *Zhonghua Gan Zang Bing Za Zhi* 2008, **16**(3):207-209.

37. Jin WD, Chen LH, Mu F: **[Enhancement of HepG2 cell radiosensitivity by mutant IkappaBalpha gene transfection]**. *Nan Fang Yi Ke Da Xue Xue Bao* 2008, **28**(3):413-416.

38. Bai C, Yang M, Fan Z, Li S, Gao T, Fang Z: **Associations of chemo- and radio-resistant phenotypes with the gap junction, adhesion and extracellular matrix in a three-dimensional culture model of soft sarcoma**. *J Exp Clin Cancer Res* 2015, **34**(1):58.

39. Moneef MA, Sherwood BT, Bowman KJ, Kockelbergh RC, Symonds RP, Steward WP, Mellon JK, Jones GD: **Measurements using the alkaline comet assay predict bladder cancer cell radiosensitivity**. *Br J Cancer* 2003, **89**(12):2271-2276.

40. McKeown SR, Robson T, Price ME, Ho ET, Hirst DG, McKelvey-Martin VJ: **Potential use of the alkaline comet assay as a predictor of bladder tumour response to radiation**. *Br J Cancer* 2003, **89**(12):2264-2270.

41. Roos WP, Binder A, Böhm L: **Determination of the initial DNA damage and residual DNA damage remaining after 12 hours of repair in eleven cell lines at low doses of irradiation**. *International journal of radiation biology* 2000, **76**(11):1493-1500.

42. Schwarz SB, Schaffer PM, Kulka U, Ertl-Wagner B, Hell R, Schaffer M: **The effect of radio-adaptive doses on HT29 and GM637 cells**. *Radiat Oncol* 2008, **3**:12.

43. Livingstone A, Mairs RJ, Russell J, O'Donoghue J, Gaze MN, Wheldon TE: **N-myc gene copy number in neuroblastoma cell lines and resistance to experimental treatment**. *European journal of cancer (Oxford, England : 1990)* 1994, **30a**(3):382-389.

44. Price ME, McKelvey-Martin VJ, Robson T, Hirst DG, McKeown SR: **Induction and rejoining of DNA double-strand breaks in bladder tumor cells**. *Radiation research* 2000, **153**(6):788-794.

45. Akudugu JM, Böhm L: **Micronuclei and apoptosis in glioma and neuroblastoma cell lines and role of other lesions in the reconstruction of cellular radiosensitivity**. *Radiation and environmental biophysics* 2001, **40**(4):295-300.

46. Wang JL, Yu JP, Sun ZQ, Sun SP: **Radiobiological characteristics of cancer stem cells from esophageal cancer cell lines**. *World J Gastroenterol* 2014, **20**(48):18296-18305.

47. Liu Q, Jiang H, Liu Z, Wang Y, Zhao M, Hao C, Feng S, Guo H, Xu B, Yang Q *et al*: **Berberine radiosensitizes human esophageal cancer cells by downregulating homologous recombination repair protein RAD51**. *PloS one* 2011, **6**(8):e23427.

48. Ruhl R, Rana S, Kelley K, Espinosa-Diez C, Hudson C, Lanciault C, Thomas CR, Jr., Liana Tsikitis V, Anand S: **microRNA-451a regulates colorectal cancer proliferation in response to radiation**. 2018, **18**(1):517.

49. Gigante M, Toffoli G, Bertola A, Biscontin G, Dassie A, Zanelli GD, Zanin E, Trovò MG, Muzzio PC: **Radiosensitivity in multidrug-resistant and cisplatin-resistant human carcinoma cell lines**. *Am J Clin Oncol* 2003, **26**(4):e73-79.

50. Suardet L, Li C, Little JB: **Radio-induced modulation of transforming growth factor beta1 sensitivity in a p53 wild-type human colorectal-cancer cell line**. *International journal of cancer* 1996, **68**(1):126-131.

51. Allalunis-Turner MJ, Zia PK, Barron GM, Mirzayans R, Day RS, 3rd: **Radiation-induced DNA damage and repair in cells of a radiosensitive human malignant glioma cell line**. *Radiation research* 1995, **144**(3):288-293.

52. Li P, Zhang Q, Torossian A, Li ZB, Xu WC, Lu B, Fu S: **Simultaneous inhibition of EGFR and PI3K enhances radiosensitivity in human breast cancer**. *International journal of radiation oncology, biology, physics* 2012, **83**(3):e391-397.

53. Dunne AL, Price ME, Mothersill C, McKeown SR, Robson T, Hirst DG: **Relationship between clonogenic radiosensitivity, radiation-induced apoptosis and DNA damage/repair in human colon cancer cells**. *Br J Cancer* 2003, **89**(12):2277-2283.

54. Lambin P, Malaise EP, Joiner MC: **Might intrinsic radioresistance of human tumour cells be induced by radiation?** *International journal of radiation biology* 1996, **69**(3):279-290.

55. Lambin P, Coco-Martin J, Legal JD, Begg AC, Parmentier C, Joiner MC, Malaise EP: **Intrinsic radiosensitivity and chromosome aberration analysis using fluorescence in situ hybridization in cells of two human tumor cell lines**. *Radiation research* 1994, **138**(1 Suppl):S40-43.

56. Russell JS, Lang FF, Huet T, Janicot M, Chada S, Wilson DR, Tofilon PJ: **Radiosensitization of human tumor cell lines induced by the adenovirus-mediated expression of an anti-Ras single-chain antibody fragment**. *Cancer research* 1999, **59**(20):5239-5244.

57. Gao Z, Zhuang L, Chen Y: **[Effect and mechanism of gefitinib inhibition on non-small cell lung cancer radiosensitivity of HCC827 and H358 cell lines]**. *Zhongguo fei ai za zhi = Chinese journal of lung cancer* 2012, **15**(6):324-331.

58. Kimura H: **[Usefulness of micronucleus assay in radiosensitivity tests using human cancer cell lines]**. *Nihon Hinyokika Gakkai Zasshi* 1998, **89**(8):712-720.

59. Wang ZM, Lu J, Zhang LY, Lin XZ, Chen KM, Chen ZJ, Liu FJ, Yan FH, Teng GJ, Mao AW: **Biological effects of low-dose-rate irradiation of pancreatic carcinoma cells in vitro using 125I seeds**. *World J Gastroenterol* 2015, **21**(8):2336-2342.

60. Huang Y, Yu J, Yan C, Hou J, Pu J, Zhang G, Fu Z, Wang X: **Effect of small interfering RNA targeting hypoxia-inducible factor-1α on radiosensitivity of PC3 cell line**. *Urology* 2012, **79**(3):744.e717-724.

61. Scott SL, Gumerlock PH, Beckett L, Li Y, Goldberg Z: **Survival and cell cycle kinetics of human prostate cancer cell lines after single- and multifraction exposures to ionizing radiation**. *International journal of radiation oncology, biology, physics* 2004, **59**(1):219-227.

62. Yu ZQ, Zhang C, Lao XY, Wang H, Gao XH, Cao GW, Zhou WP, Fu CG: **[Long non-coding RNA influences radiosensitivity of colorectal carcinoma cell lines by regulating cyclin D1 expression]**. *Zhonghua Wei Chang Wai Ke Za Zhi* 2012, **15**(3):288-291.

63. Liu ZG, Chen HY, Cheng JJ, Chen ZP, Li XN, Xia YF: **Relationship between methylation status of ERCC1 promoter and radiosensitivity in glioma cell lines**. *Cell Biol Int* 2009, **33**(10):1111-1117.

64. Moiseenko V, Banáth JP, Duzenli C, Olive PL: **Effect of prolonging radiation delivery time on retention of gammaH2AX**. *Radiat Oncol* 2008, **3**:18.

65. Wilkins RC, Ng CE, Raaphorst GP: **Comparison of high dose rate, low dose rate, and high dose rate fractionated radiation for optimizing differences in radiosensitivities in vitro**. *Radiat Oncol Investig* 1998, **6**(5):209-215.

66. Akudugu JM, Slabbert JP, Serafin A, Bohm L: **Frequency of radiation-induced micronuclei in neuronal cells does not correlate with clonogenic survival**. *Radiation research* 2000, **153**(1):62-67.

67. Jiang YH, You KY, Bi ZF, Li LT, Mo HQ, Liu YM: **[The relationship between the radioresistance of pancreatic cancer cell SW1990 and the induction of the Epithelial-Mesenchymal Transition: an in vitro study]**. *Zhonghua Yi Xue Za Zhi* 2018, **98**(12):939-943.

68. Wen Q, Zhao YP, Chen G, Zhang FQ, Zhang TP: **[Expression of S100A2 in secondarily radioresistant pancreatic cancer cells and significance thereof]**. *Zhonghua Yi Xue Za Zhi* 2006, **86**(40):2817-2820.

69. Gupta AK, Bernhard EJ, Bakanauskas VJ, Wu J, Muschel RJ, McKenna WG: **RAS-Mediated radiation resistance is not linked to MAP kinase activation in two bladder carcinoma cell lines**. *Radiation research* 2000, **154**(1):64-72.

70. Rouhani M, Goliaei B, Khodagholi F, Nikoofar A: **Lithium increases radiosensitivity by abrogating DNA repair in breast cancer spheroid culture**. *Arch Iran Med* 2014, **17**(5):352-360.

71. Murad H, Alghamian Y, Aljapawe A, Madania A: **Effects of ionizing radiation on the viability and proliferative behavior of the human glioblastoma T98G cell line**. *BMC Res Notes* 2018, **11**(1):330.

72. Hu L, Wu QQ, Wang WB, Jiang HG, Yang L, Liu Y, Yu HJ, Xie CH, Zhou YF, Zhou FX: **Suppression of Ku80 correlates with radiosensitivity and telomere shortening in the U2OS telomerase-negative osteosarcoma cell line**. *Asian Pac J Cancer Prev* 2013, **14**(2):795-799.

73. Mirjolet C, Papa AL, Créhange G, Raguin O, Seignez C, Paul C, Truc G, Maingon P, Millot N: **The radiosensitization effect of titanate nanotubes as a new tool in radiation therapy for glioblastoma: a proof-of-concept**. *Radiotherapy and oncology : journal of the European Society for Therapeutic Radiology and Oncology* 2013, **108**(1):136-142.

74. Cerami E, Gao J, Dogrusoz U, Gross BE, Sumer SO, Aksoy BA, Jacobsen A, Byrne CJ, Heuer ML, Larsson E *et al*: **The cBio cancer genomics portal: an open platform for exploring multidimensional cancer genomics data**. *Cancer Discov* 2012, **2**(5):401-404.

75. Colaprico A, Silva TC, Olsen C, Garofano L, Cava C, Garolini D, Sabedot TS, Malta TM, Pagnotta SM, Castiglioni I *et al*: **TCGAbiolinks: an R/Bioconductor package for integrative analysis of TCGA data**. *Nucleic acids research* 2016, **44**(8):e71.

76. Goldman MJ, Craft B, Hastie M, Repečka K, McDade F, Kamath A, Banerjee A, Luo Y, Rogers D, Brooks AN *et al*: **Visualizing and interpreting cancer genomics data via the Xena platform**. *Nature biotechnology* 2020, **38**(6):675-678.

77. Rau A, Flister M, Rui H, Auer PL: **Exploring drivers of gene expression in the Cancer Genome Atlas**. *Bioinformatics (Oxford, England)* 2019, **35**(1):62-68.

78. Mermel CH, Schumacher SE, Hill B, Meyerson ML, Beroukhim R, Getz G: **GISTIC2.0 facilitates sensitive and confident localization of the targets of focal somatic copy-number alteration in human cancers**. *Genome biology* 2011, **12**(4):R41.

79. McKenna A, Hanna M, Banks E, Sivachenko A, Cibulskis K, Kernytsky A, Garimella K, Altshuler D, Gabriel S, Daly M *et al*: **The Genome Analysis Toolkit: a MapReduce framework for analyzing next-generation DNA sequencing data**. *Genome Res* 2010, **20**(9):1297-1303.

80. Katzman JL, Shaham U, Cloninger A, Bates J, Jiang T, Kluger Y: **DeepSurv: personalized treatment recommender system using a Cox proportional hazards deep neural network**. *BMC Medical Research Methodology* 2018, **18**(1):24.

81. Ko D: **Applied survival analysis: regression modeling of time to event data: David W. Hosmer Jr. and Stanley Lemeshow, Wiley, New York, 1999, pp. 386+xiii price $84.95 ISBN 0-471-15410-5**. *Journal of Statistical Planning and Inference* 2000, **91**(1):173-175.

82. Knaus WA, Harrell FE, Jr., Lynn J, Goldman L, Phillips RS, Connors AF, Jr., Dawson NV, Fulkerson WJ, Jr., Califf RM, Desbiens N *et al*: **The SUPPORT prognostic model. Objective estimates of survival for seriously ill hospitalized adults. Study to understand prognoses and preferences for outcomes and risks of treatments**. *Ann Intern Med* 1995, **122**(3):191-203.

83. Curtis C, Shah SP, Chin S-F, Turashvili G, Rueda OM, Dunning MJ, Speed D, Lynch AG, Samarajiwa S, Yuan Y *et al*: **The genomic and transcriptomic architecture of 2,000 breast tumours reveals novel subgroups**. *Nature* 2012, **486**(7403):346-352.

84. Schumacher M, Bastert G, Bojar H, Hübner K, Olschewski M, Sauerbrei W, Schmoor C, Beyerle C, Neumann RL, Rauschecker HF: **Randomized 2 x 2 trial evaluating hormonal treatment and the duration of chemotherapy in node-positive breast cancer patients. German Breast Cancer Study Group**. *Journal of clinical oncology : official journal of the American Society of Clinical Oncology* 1994, **12**(10):2086-2093.

85. Shedden K, Taylor JM, Enkemann SA, Tsao MS, Yeatman TJ, Gerald WL, Eschrich S, Jurisica I, Giordano TJ, Misek DE *et al*: **Gene expression-based survival prediction in lung adenocarcinoma: a multi-site, blinded validation study**. *Nature medicine* 2008, **14**(8):822-827.

86. Gautier L, Cope L, Bolstad BM, Irizarry RA: **affy--analysis of Affymetrix GeneChip data at the probe level**. *Bioinformatics (Oxford, England)* 2004, **20**(3):307-315.

87. Kosmidis I, Firth D: **A generic algorithm for reducing bias in parametric estimation**. *Electronic Journal of Statistics* 2010, **4**.

88. Ashburner M, Ball CA, Blake JA, Botstein D, Butler H, Cherry JM, Davis AP, Dolinski K, Dwight SS, Eppig JT *et al*: **Gene Ontology: tool for the unification of biology**. *Nature Genetics* 2000, **25**(1):25-29.

89. Kim M-S, Kim W, Park IH, Kim HJ, Lee E, Jung J-H, Cho LC, Song CW: **Radiobiological mechanisms of stereotactic body radiation therapy and stereotactic radiation surgery**. *Radiation oncology journal* 2015, **33**(4):265-275.

90. Yu G, Wang LG, Han Y, He QY: **clusterProfiler: an R package for comparing biological themes among gene clusters**. *Omics : a journal of integrative biology* 2012, **16**(5):284-287.

91. Andersen PK, Gill RD: **Cox's Regression Model for Counting Processes: A Large Sample Study**. *The Annals of Statistics* 1982, **10**(4):1100-1120.

92. Wilkinson GN, Rogers CE: **Symbolic Description of Factorial Models for Analysis of Variance**. *Journal of the Royal Statistical Society: Series C (Applied Statistics)* 1973, **22**(3):392-399.

93. Chih-Chung C, Chih-Jen L: **Libsvm: a library for support vector machines**. *ACM Trans Intell Syst Technol, vol 2(3)* 2011:1-27.

94. Breiman L: **Random Forests**. *Machine Learning* 2001, **45**(1):5-32.

95. Ishwaran H, Kogalur U: **randomForestSRC: Random Forests for Survival, Regression and Classification (RF-SRC)**. 2016.

96. Eschrich S, Zhang H, Zhao H, Boulware D, Lee JH, Bloom G, Torres-Roca JF: **Systems biology modeling of the radiation sensitivity network: a biomarker discovery platform**. *International journal of radiation oncology, biology, physics* 2009, **75**(2):497-505.

97. Gaujoux R, Seoighe C: **A flexible R package for nonnegative matrix factorization**. *BMC bioinformatics* 2010, **11**:367.

98. Hartigan JA, Wong MA: **A K-means clustering algorithm**. *Applied Statistics* 2013, **28**:100-108.

99. Ritchie ME, Phipson B, Wu D, Hu Y, Law CW, Shi W, Smyth GK: **limma powers differential expression analyses for RNA-sequencing and microarray studies**. *Nucleic acids research* 2015, **43**(7):e47.

100. Langfelder P, Horvath S: **WGCNA: an R package for weighted correlation network analysis**. *BMC bioinformatics* 2008, **9**:559.

101. Szklarczyk D, Morris JH, Cook H, Kuhn M, Wyder S, Simonovic M, Santos A, Doncheva NT, Roth A, Bork P *et al*: **The STRING database in 2017: quality-controlled protein-protein association networks, made broadly accessible**. 2017, **45**(D1):D362-d368.

102. Wen P, Xia J, Cao X, Chen B, Tao Y, Wu L, Xu A, Zhao G: **dbCRSR: a manually curated database for regulation of cancer radiosensitivity**. *Database (Oxford)* 2018, **2018**.

103. Cox DR: **Partial likelihood**. *Biometrika* 1975, **62**(2):269-276.
